# Intensity Modulated Radiation Fields Induce Protective Effects and Reduce Importance of Dose-Rate Effects

**DOI:** 10.1038/s41598-019-45960-z

**Published:** 2019-07-01

**Authors:** Yusuke Matsuya, Stephen J. McMahon, Mihaela Ghita, Yuji Yoshii, Tatsuhiko Sato, Hiroyuki Date, Kevin M. Prise

**Affiliations:** 1 Japan Atomic Energy Agency (JAEA), Nuclear Science and Engineering Center, Research Group for Radiation Transport Analysis, 2-4 Shirakata, Tokai, 319-1195 Ibaraki Japan; 2 Graduate School of Health Sciences, Hokkaido University, Kita-12 Nishi-5, Kita-ku, Sapporo, 060-0812 Hokkaido Japan; 3 Centre for Cancer Research and Cell Biology, Queen’s University Belfast, 97 Lisburn Road, BT7 9AE Belfast, UK; 4 Biological Research, Education and Instrumentation Center, Sapporo Medical University, Minami-1 Nishi-17, Chuo-ku, Sapporo, 060-8556 Hokkaido Japan; 5 Faculty of Health Sciences, Hokkaido University, Kita-12 Nishi-5, Kita-ku, Sapporo, 060-0812 Hokkaido Japan

**Keywords:** Apoptosis, Applied mathematics, Computational science

## Abstract

In advanced radiotherapy, intensity modulated radiation fields and complex dose-delivery are utilized to prescribe higher doses to tumours. Here, we investigated the impact of modulated radiation fields on radio-sensitivity and cell recovery during dose delivery. We generated experimental survival data after single-dose, split-dose and fractionated irradiation in normal human skin fibroblast cells (AGO1522) and human prostate cancer cells (DU145). The dose was delivered to either 50% of the area of a T25 flask containing the cells (half-field) or 100% of the flask (uniform-field). We also modelled the impact of dose-rate effects and intercellular signalling on cell-killing. Applying the model to the survival data, it is found that (i) in-field cell survival under half-field exposure is higher than uniform-field exposure for the same delivered dose; (ii) the importance of sub-lethal damage repair (SLDR) in AGO1522 cells is reduced under half-field exposure; (iii) the yield of initial DNA lesions measured with half-field exposure is smaller than that with uniform-field exposure. These results suggest that increased cell survival under half-field exposure is predominantly attributed not to rescue effects (increased SLDR) but protective effects (reduced induction of initial DNA lesions). In support of these protective effects, the reduced DNA damage leads to modulation of cell-cycle dynamics, i.e., less G_1_ arrest 6 h after irradiation. These findings provide a new understanding of the impact of dose-rate effects and protective effects measured after modulated field irradiation.

## Introduction

Recent radiotherapy has evolved to use modulated radiation intensity and complex dose-delivery techniques such as intensity-modulated radiation therapy (IMRT) and volumetric modulated arc therapy (VMAT). These approaches provide a high level of dose conformity to the target tumour volume, preserving organs at risk^1,2^. Dose-rates used for external irradiation in radiotherapy (i.e., about 4 Gy/min)^3^ are much higher than brachytherapy (i.e., about 12 Gy/h or 50 cGy/h)^4^, however some modalities such as IMRT, MRI-linacs^5^ and Cyberknife^6^ need relatively long times to deliver the prescribed dose compared to previously used methods such as conformal therapy (3D-CRT)^7^. Recent clinical dose-rate studies indicate that cell recovery during irradiation (hereafter called sub-lethal damage repair: SLDR^8,9^) cannot be ignored, even for the case of high-dose-rate radiation therapy^10^.

Radiation-hit cells and non-hit cells co-exist during intensity modulated field exposures. Regarding this, *in vitro* experimental configurations containing in-field and out-of-field cells have been established^11^, and their biological effects have been previously studied^11–14^. Specifically, it was shown that using a 50% in-field and 50% out-of-field (half-field) irradiation as a simple model of modulated-field treatment, intercellular communication (IC) from cells in-field to cells out-of-field reduces survival of out-of-field cells^11,15^. This enhancement of cell death attributed to IC is referred to non-targeted effects or radiation-induced bystander effects^16–20^. In contrast, there are also several reports about signal-induced radio-resistance^21–23^, which can sometimes be observed in cells in-field under half-field irradiation in comparison with a uniform field exposure^12,13^. This radio-resistance is assumed to be attributed to the increase of DNA repair efficiency by rescue effects^23^. However, McGarry *et al*. showed that there was no statistically significant cell recovery of AGO-1522b cells during the delivery of a 9-field IMRT delivery in comparison to acute irradiation at 4 Gy/min^1^. This response contradicts what is expected from reported rescue effects. Thus, we are interested in elucidating the underlying mechanisms of modulated beam exposures via a comprehensive analysis of *in vitro* experiments combined with modelling approaches.

From the standpoint of modelling studies, the linear-quadratic (LQ) model^24,25^ has been generally accepted in the fields of radiation therapy and radiation biology^26,27^. However, more detailed models are needed to define mechanisms by considering effects due to microdosimetry and cell recovery by virtue of SLDR^28–31^. For example, the time factor in the microdosimetric-kinetic (MK) model^28^ represents the sub-lethal damage repair (SLDR) rate which can be deduced from a split-dose cell recovery curve^30^. Amongst many models developed by several researchers^15,28–33^, the “*integrated microdosimetric-kinetic* (*IMK*) *model*”^34,35^ is suitable for analysing impact of IC for intensity-modulated fields and SLDR during irradiation. From *in vitro* experiments for modulated fields, we have also used this modelling approach to interpret the mechanisms of the radio-resistance.

Here, we focused on radio-sensitivity and dose-rate effects following exposure to intensity modulated fields. Using a simple geometry where 50% of the area of the cell culture flask is exposed, the in-field cell survival and out-of-field cell survival were quantified. Through this comprehensive study with *in vitro* experiments and modelling, we show the reduced importance of SLDR and presence of protective effects in irradiated healthy cells in modulated fields.

## Materials and Methods

### Cell culture

Experiments were performed using two human cell lines, the human skin fibroblast cell line, AGO1522, as a normal cell model, and the human prostate cancer cell line, DU145, as a tumour cell model. AGO1552 and DU145 cells were obtained from the Coriell Institute for Medical Research (Camden, NJ, USA) and Cancer Research UK, respectively. AGO1522 cells were grown in Eagle’s minimum essential medium supplemented with 20% fetal bovine serum (FBS) and 1% penicillin/streptomycin (p/s). DU145 cells were grown in RPMI-1640 with L-glutamine supplemented with 10% FBS, 1% p/s. These cell lines were maintained at 37 °C in a humidified atmosphere of 5% CO_2_.

### Irradiation setup and planning

All irradiations in this study have been performed using a 225 kVp X-ray (Precision x-Ray) source at dose rates of 0.59 Gy/min or 0.18 Gy/min. The dose was delivered to either 50% of the area of T25 flask containing cells or 100% of the flask as previously reported^11^.

**Figure 1:**
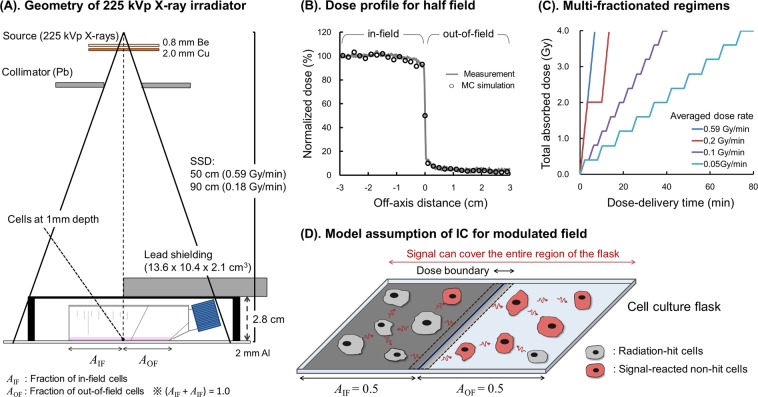
Study design for *in vitro* experiments with irradiation, Monte Carlo simulation and assumptions of the mathematical model: (**A**) illustrates the geometry of half-field irradiation, (**B**) is the dose profile compared between Gafchromic film and Monte Carlo simulation by PHITS ver. 3.02^36^, (**C**) is the temporal characteristics of dose delivered to cultured cells at different dose-rates and (**D**) represents the assumptions for modelling the signal range under half-field exposure, where *A*_IF_ = 0.5 (gray area) and *A*_OF_ = 0.5 (light gray area) are the fractions of in-field and out-of-field area in the modulated field, respectively. It is assumed that intercellular signals can cover the entire region of the flask for both cases of half and uniform-fields, based on previous experimental data^14^.

For the exposure of 50% cells in a culture flask, a T25 flask (Nunclon surface NUNC) was placed at the center of radiation beam, and half of the flask was shielded using a lead block (13.6 × 10.4 × 2.1 cm^3^ lead blocks MCP60-Mining & Chemical Products Ltd.). At the bottom of each flask, RTQA Gafchromic® film (Vertec Scientific Ltd.) was attached to monitor the dose boundaries. Schematic representations of the irradiation geometry and dose profile are illustrated in Fig. 1A,B. The dose profile was also checked by using the Monte Carlo simulation code, Particle and Heavy Ion Transport Code System (PHITS ver. 3.02)^36^.

The cells were exposed by means of either a single-dose irradiation, a split-dose irradiation to evaluate sub-lethal damage repair (SLDR) or one of four different dose regimen for the dose-rate study. For split-dose experiments, a dose of 4 Gy split into two equal fractions was used with various inter-fraction times (0, 0.25, 0.5, 1, 2, 4, 24 and 48 h). We also compared four different dose plans equivalent to 0.59, 0.2, 0.1, 0.05 Gy/min, on average, for a total absorbed dose of 4 Gy. A temporal cell’s-eye view of dose-delivery is described in Fig. 1C.

### Clonogenic survival assay

Cell survival was measured by means of a clonogenic assay as previously described^11^. Cells were plated and allowed to adhere overnight before irradiation. All exposures were performed at room temperature. The cells, after irradiation, were incubated for 10–14 days before staining with 0.5% crystal violet in 70% methanol.

### Flow cytometric analysis of cell-cycle distribution

To understand changes in radio-sensitivity under half-field exposure, we also measured cell-cycle dynamics by using a co-culture system. Co-cultured cells were plated sub-confluently on glass slides and allowed to adhere overnight as previously reported^37^.

Less than 1 × 10^6^ cells at 0, 6, 24, 72 h after irradiation were fixed with 70% ethanol (in 0.5 mL of PBS and 4.5 mL of 70% ethanol) and kept at 4 °C for at least 2 h. After centrifugation, the cells were suspended in 1 mL PBS. After centrifugation for 10 min at 300 g at 4 °C, ethanol was thoroughly removed (or decanted). Cells were then treated with 0.5 mL of PI staining solution (20 μg/mL PI, 20 μg/mL DNase-free RNase A, 0.1%v/v TritonX100) and kept in the dark at 37 °C for 15 min.

### Model analysis and overview

A mathematical model was used to analyse the measured experimental data^34,35^. Cell survival considering DNA damage along the radiation track (so called DNA targeted effects: DNA-TEs) and Non-Targeted Effects (NTEs) was previously modelled using the IMK model^34^. In this study, the IMK model was modified so as to include the modulated radiation field shown in Fig. 1A. In this model, reparable lesions (potentially lethal lesion: PLL) are assumed to be DNA lesions with cell toxicity, which may transform into non-reparable lesions (lethal lesions: LLs) or be completely repaired^28^. Solving the kinetic equation for DNA lesions, cell surviving fraction can be calculated for both half-field (*A*_IF_ = 0.5) and uniform-field (*A*_IF_ = 1.0) cases, where *A*_IF_ is the fraction of in-field area used in the experimental design (Fig. 1A).

### Modelling of targeted effects (TEs)

The conventional MK model for continuous irradiation which considers discontinuous energy deposition to micrometre targets (or domains) is used for describing cell killing induced by DNA-TEs^31,35^, where multi-fractionated exposures to cell population with a dose-delivery time *T* (h) and *N* dose fractions are assumed. Based on previous modelling^10,35^, the cell surviving fraction for DNA-TEs *S*_T_ can be given by:

1 $$\begin{array}{rcl}-\,\mathrm{ln}\,{S}_{{\rm{T}}} & = & {\sum }_{n=1}^{N}\,[({\alpha }_{0}\,+\,\gamma {\beta }_{0}){D}_{n}\Delta T\,+\,{\beta }_{0}{({D}_{n}\Delta T)}^{2}]\\  &  & +2{\sum }_{n=1}^{N-1}\,{\sum }_{m=n+1}^{N}\,[{\beta }_{0}\langle {e}^{-(m-n)(a+c)\Delta T}\rangle {D}_{n}{D}_{m}]\Delta {T}^{2}.\end{array}$$

where *ΔT* is a sub-section of *T* defined by *T* = *NΔT*, *D*_*n*_ is dose delivered to cell population in the period of *ΔT*, *α*_0_ and *β*_0_ are the cell-specific coefficients for dose (Gy) and dose squared (Gy^2^), respectively; (*a* + *c*) is sum of constant rates for a PLL to transform into a LL and to be repaired (h^−1^), representing approximately SLDR due to the relation of (*a* + *c*) $\cong$ *c*^30,38^; *γ* (Gy) is the microdosimetric quantity expressed by *γ* = *y*_*D*_/(*ρπr*_d_^2^); *ρ* and *r*_d_ represent the density of liquid water (1.0 g/cm^3^) and radius of a domain^39^ (set as 0.5 μm in this study); *y*_*D*_ is dose-mean lineal energy in keV/μm^29^.

Taking a limit of *N* to infinity, the surviving fraction for the continuous irradiation case, with a constant dose-rate $\dot{D}$ (Gy/h), can be expressed by using the Lea-Catcheside time factor *F* as follows:

2 $$\begin{array}{c}-\,\mathrm{ln}\,{S}_{{\rm{T}}}=({\alpha }_{0}+\gamma {\beta }_{0})\dot{D}T+\frac{2{\beta }_{0}}{{(a+c)}^{2}{T}^{2}}[(a+c)T+{e}^{-(a+c)T}-1]{(\dot{D}T)}^{2}\\ \,\,\,\,=({\alpha }_{0}+\gamma {\beta }_{0})D+F{\beta }_{0}{D}^{2}\end{array}$$

where

3a $$F=\frac{2}{{(a+c)}^{2}{T}^{2}}[(a+c)T+{e}^{-(a+c)T}-1],$$

3b $$D=\dot{D}T,$$

where *D* is absorbed dose delivered to the cell population during the irradiation. In the model for DNA-targeted effects, (*a* + *c*) and *β*_0_ can be obtained from a split dose cell recovery curve, whilst *α*_0_ can be determined by fitting to the acute exposure cell survival curve.

### Modelling of intercellular communication (IC)

The modelling of intercellular signalling between in-field and out-of-field cells is based on the IMK model, which incorporates the kinetics of signal concentration emitted from irradiated cells, DNA repair kinetics and cell survival^34^. In the case of the half-field exposures considered here (*A*_IF_ = 0.5 as shown in Fig. 1A), we assume that the intercellular signals from in-field cells cover the entire out-of-field region (Fig. 1D), based on previous experimental data^14^. Concerning the time course of dose-delivery, the out-of-field surviving fraction appears to be independent of dose fractionation^13^. So here, we made the following assumptions for NTEs:

(i) Targets of micron-order size, which trigger the release of intercellular signals, exist somewhere within a cell, for example, mitochondria^40^. Intercellular cell-killing signals are emitted from target-activated cells (hit cells), and interact with cells without any activated targets (non-hit cells) in the flask. (Fig. 1D). The mean number of targets activated for releasing IC signals per hit cell is defined as ($({\alpha }_{{\rm{b}}}+\gamma {\beta }_{{\rm{b}}})D+{\beta }_{{\rm{b}}}{D}^{2}$), where *α*_b_ and *β*_b_ are coefficients for *D* and *D*^2^ for NTEs, respectively.
(ii) The probability of a given cell having an activated target for emitting NTE signals *f*_h_(*D*) follows Poisson statistics with the number of activated targets for NTEs, giving *f*_h_(*D*) = 1−${e}^{-[({\alpha }_{{\rm{b}}}+\gamma {\beta }_{{\rm{b}}})D+{\beta }_{{\rm{b}}}{D}^{2}]}$^34^, where *D* denotes cumulative absorbed dose. The probability of a cell having no activated targets can be deduced as 1−*f*_h_(*D*). *f*_h_(*D*) and *f*_b_(*D*) = ${e}^{-[({\alpha }_{{\rm{b}}}+\gamma {\beta }_{{\rm{b}}})D+{\beta }_{{\rm{b}}}{D}^{2}]}$. This is taken to be independent of dose fractionation according to the experimental data from split-dose irradiation^12^.
(iii) To account for the generation of cell-killing signals, their decay and reaction with non-hit cells^34,41^, it is assumed that the yield of lethal lesions (LLs) in non-hit cells is proportional to the parameter *δ*^34^, which is determined empirically.

When delivering dose *D* (Gy) to the in-field region, the hit probability for in-field irradiated cells *f*_h_(*D*)_IF_ is equal to $1-{e}^{-({\alpha }_{{\rm{b}}}+{\gamma }_{{\rm{I}}{\rm{F}}}{\beta }_{{\rm{b}}}){D}_{{\rm{I}}{\rm{F}}}-{\beta }_{{\rm{b}}}{{D}_{{\rm{I}}{\rm{F}}}}^{2}}$. The mean number of LLs per cells induced by NTEs *w*_NT_, due to the effect of cell-killing signals from in-field area in the out-of-field area, can be expressed by using *f*_h_(*D*), *f*_b_(*D*) and *δ* as follows:

4 $${w}_{{\rm{NT}}}=\delta \,{f}_{{\rm{h}}}{(D)}_{{\rm{IF}}}\,{f}_{{\rm{b}}}{(D)}_{\ast }$$

where *f*_b_(*D*)_*_ is the fraction of non-hit cells either in-field or out-of-field area (the symbol * stands for either in-field (IF) or out-of-field (OF)). Assuming Poisson statistics for the number of LLs per nucleus created by NTEs, the cell survival for NTEs can be expressed as

5 $$-\,{\rm{l}}{\rm{n}}\,{S}_{{\rm{N}}{\rm{T}}}=\delta [1-\,{e}^{-({\alpha }_{{\rm{b}}}+{\gamma }_{{\rm{I}}{\rm{F}}}{\beta }_{{\rm{b}}}){D}_{{\rm{I}}{\rm{F}}}-{\beta }_{{\rm{b}}}{{D}_{{\rm{I}}{\rm{F}}}}^{2}}]{e}^{-({\alpha }_{{\rm{b}}}+{\gamma }_{\ast }{\beta }_{{\rm{b}}}){D}_{\ast }-{\beta }_{{\rm{b}}}{{D}_{\ast }}^{2}}$$

where *S*_NT_ is the surviving fraction of cells available for IC. The set of model parameters *α*_b_, *β*_b_ and *δ* are cell-specific and can be determined from fits to the experimental out-of-field cell survival curves.

### Cell surviving fraction considering TEs and NTE for modulated radiation fields

Here, we stochastically consider the fraction of cells with which intercellular signals react, and from which the integrated surviving fraction for TEs and NTEs is deduced.

Assuming that the interaction probability between sub-lesions (PLLs) in TEs and NTEs is very small^34,42^, the total number of LLs per nucleus considering TEs and NTEs (*w*) can be expressed by

6 $$w={w}_{{\rm{T}}}+{w}_{{\rm{NT}}}=-\,\mathrm{ln}\,{S}_{\ast }$$

where S_*_ is the surviving fraction of cells either in-field (*S*_IF_) or out-of-field(*S*_OF_). In Eq. (6), it should be noted that the surviving fraction of cells for both half-field (*A*_IF_ = 0.5) and uniform-field (*A*_IF_ = 1.0) exposures is composed of the multiplication of survival for TEs and NTEs^34,43^.

### Model application and estimation of cell survival

To determine the model parameters in the present model, we took 3 steps as follows: (i) calculation of *y*_*D*_ values by Monte Carlo simulation, (ii) determination of SLDR rate (*a* + *c*) and *β*_0_ from a split-dose cell recovery curve, (iii) fitting of the model to dose-response curves to determine the rest of the model parameters via Markov chain Monte Carlo (MCMC). We then estimated the surviving fraction by using the obtained model parameters.

### Monte carlo simulation to calculate *y*_*D*_ value

We obtained *y*_*D*_ values for 225 kVp X-rays by using two Monte Carlo simulation codes, PHITS^36^ and WLTrack^44^. The geometry is illustrated in Fig. 1A, which is validated by comparing the dose profile measured by Gafchromic film with that calculated by the PHITS simulation (Fig. 1B). The detail of geometry for calculating the *y*_*D*_ value is summarized in Supplementary Information I (*“Monte Carlo Simulation for y*_*D*_ *Calculation”*).

The calculated *y*_*D*_ values were 4.393 ± 0.007 keV/μm for the in-field area and 4.769 ± 0.044 keV/μm for the out-of-field area. These values were converted to *γ* values and used as input parameters to describe the cell surviving fraction for each field area.

### Deduction of SLDR Rate from split-dose cell recovery

According to previous reports of the MK model^30^, the formula to calculate the (*a* + *c*) value, that is approximately equivalent to SLDR, is given by

7 $$(a+c)=\frac{\mathop{\mathrm{lim}}\limits_{\tau \to 0}\frac{1}{S}\frac{{\rm{d}}S}{{\rm{d}}\tau }\,}{\mathrm{ln}\,\frac{S(\infty )}{S(0)}}$$

where *τ* is inter-fraction time (incubation time between two irradiations) in hours, *S*(0) and *S*($\infty$) are surviving fractions *S*($\tau$) taking the limits of exposure interval ($\tau \,\to \,0,\,\tau \,\to \,\infty$), respectively. In this study, the initial slope d*S*/d*τ* was determined from the experimental surviving fraction by taking the gradient from 0 to 0.25 h whilst *S*(∞) was defined by the averaged survival from 6 to 48 h. The *β*_0_ value can be also determined from the ratio of *S*(0) and *S*($\infty$)^30^ as expressed by:

8 $${\beta }_{0}\,=\,\frac{1}{2{D}_{1}{D}_{2}}\,\mathrm{ln}\,\frac{S(\infty )}{S(0)}$$

where *D*_1_ and *D*_2_ represent the first dose and second dose, respectively.

Using Eqs (7) and (8), the set of model parameters involved in cell recovery between fractionated doses *θ* = [*β*_0_, (*a* + *c*)] were deduced.

### MCMC simulation

Using the obtained parameters of *θ* = [*γ*, *β*_0_, (*a* + *c*)], the rest of the cell-specific parameters in the IMK model *θ* = (*α*_0_, *α*_b_, *β*_b_, *δ*) were determined via a MCMC technique established previously^10^. The detail of the MCMC simulation is summarized in Supplementary Information II (*“Markov chain Monte Carlo for Determining Model Parameters”*). Particularly, it should be noted that the prior distribution of *θ* = (*α*_0_, *α*_b_, *β*_b_, *δ*) and the uncertainty for –ln *S* were set to follow a normal distribution.

By applying the cell survival formulae (Eqs (2), (5) and (6)) to experimental survival data after single-dose exposure (dose-response curve)^13,14^ in the MCMC simulation, we deduced the mean and standard deviation (s.d.) of the model parameters *θ* = (*α*_0_, *α*_b_, *β*_b_, *δ*).

### Estimation of cell surviving fraction

By the use of the obtained model parameters *θ* = [*α*_0_, *β*_0_, (*a* + *c*), *α*_b_, *β*_b_, *δ*] and formulae of the IMK model (Eqs (1), (2), (5) and (6)), the surviving fraction for in-field and out-of-field cells for the cases of *A*_IF_ = 0.5 and 1.0 were estimated.

The cell survival curves after single-dose exposure were described based on Eqs (2), (5) and (6), whilst the split-dose cell recovery curves, with two equal doses of 2 Gy, were described according to Eqs (1), (5) and (6). The fit quality for the fitting approach was checked by comparison with experimental survival obtained in this study and reference data^13,14^. As statistical measures, the coefficient of determination *R*^2^ and chi-square value were used, which are given by

9a $${R}^{2}={\rm{1}}-\frac{{\sum }_{i=1}^{n}\,{({\exp }_{i}-{{\rm{cal}}}_{i})}^{2}\,}{{\sum }_{i=1}^{n}\,{({\exp }_{i}- < \exp  > )}^{2}},$$

9b $${\chi }^{2}=\frac{1}{n}{\sum }_{i={\rm{1}}}^{n}\,\frac{{({\exp }_{i}-{{\rm{cal}}}_{i})}^{2}}{{\sigma }_{i\,\exp }^{2}}$$

where exp_*i*_ is measured cell survival, cal_*i*_ is cell survival calculated by the present model and *σ*_exp_ is the standard deviation of measured cell survival.

To evaluate SLDR in a modulated field, we compared the model estimation (Eqs (1), (5) and (6)) and experimental results for fractionated exposures with a total dose of 4 Gy, delivered by the 4 regimens described in Fig. 1C. For the dose response in the split-dose experiments, we also compared the model prediction (Eqs (1), (5) and (6)) with the previous results reported by Ghita, *et al*.^13^. The *R*^2^ value (Eq. (9a)) was also used to check the fit quality of the cell recovery curve after fractionated irradiation and the dose response after a split-dose irradiation.

## Results and Discussions

### Split-dose cell recovery and dose-response curve

**Figure 2:**
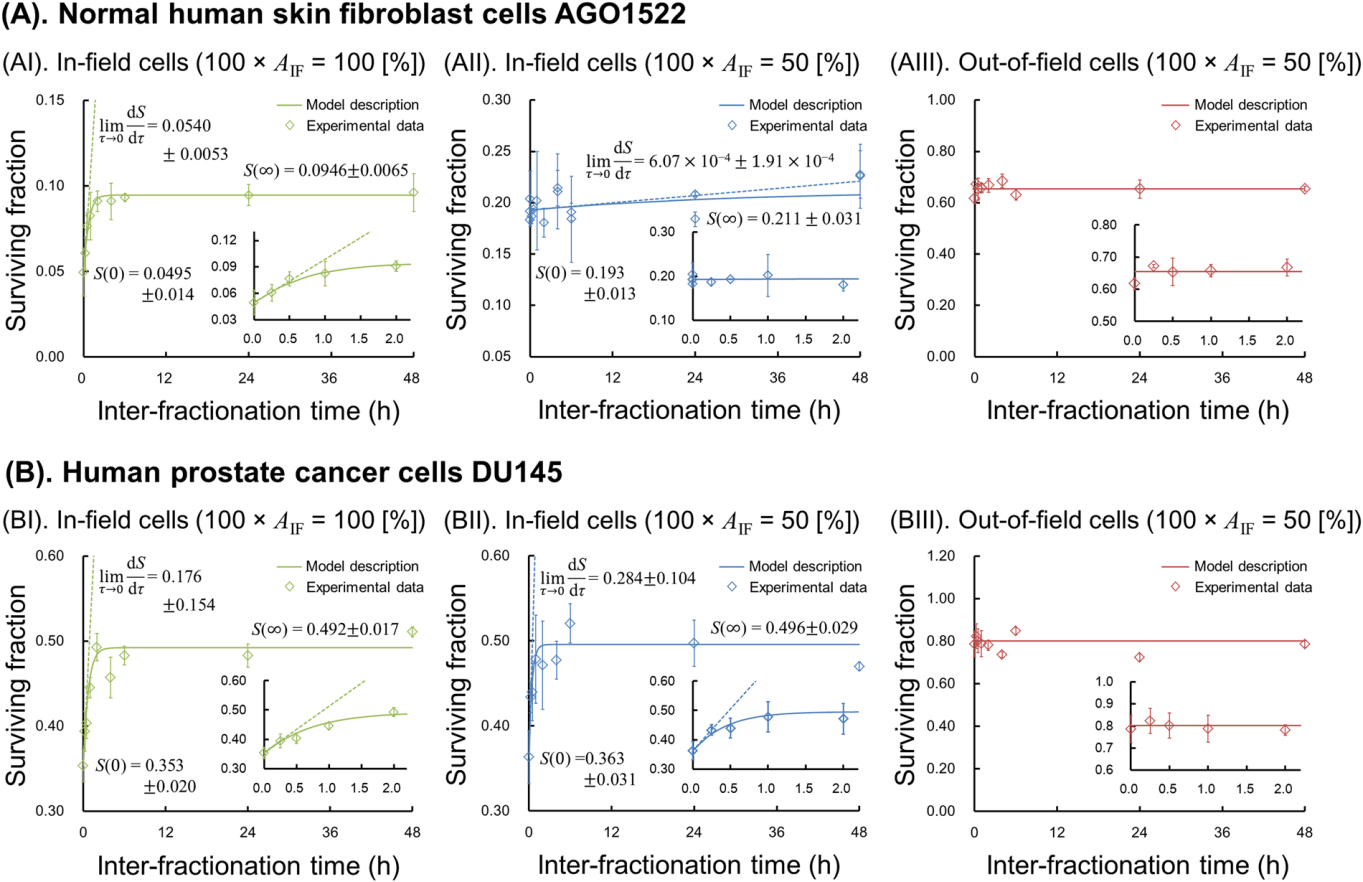
Split-dose cell recovery curves to determine sub-lethal damage repair (SLDR). Upper and lower panels are experimental cell recovery curve (symbols) and the model (solid line) for AGO1522 and DU145, respectively. Doses of 4 Gy were delivered using two equal fractions at various interfraction times (0, 0.25, 0.5, 1, 2, 4, 24, 48 h). The cell survivals of *S*(0), *S*($\infty$) and the initial slope of cell recovery [${\mathrm{lim}}_{\tau \to 0}(dS/d\tau )$] were used to determine (*a* + *c*) and *β*_0_ values based on Eqs (7) and (8). The model parameters determined by the recovery curve are listed in Table 1.

Split-dose and single-dose experiments were performed to determine the degree of SLDR and the model parameters. Figure 2 shows the cell recovery curve for survival measured by a split-dose experiment in which upper and lower panels are the results for AGO1522 and DU145, respectively. The symbols in Fig. 2 represent the experimental cell recovery. The survivals for the cases of *τ* = 0 and $\infty$ (*S*(0) and *S*($\infty$)) and the initial slop of d*S*/d*τ* (dotted line in Fig. 2) for in-field cells under uniform-field (*A*_IF_ = 1.0) (Fig. 2AI,BI) and in-field cells under half-field (*A*_IF_ = 0.5) (Fig. 2AII,BII) were used for determining the (*a* + *c*) and the *β*_0_ values based on Eqs (7) and (8). The obtained values (*a* + *c*) and *β*_0_ values are summarized in Table 1.

**Table 1:** Model parameters for modulated and uniform radiation fields.

Model parameter		Type of cell line		Unit
		AGO1522	DU145	
DNA-TEs for modulated field (MF)	*α*_0_	0.363 ± 0.013	0.032 ± 0.006	Gy^−1^
	*β*_0_	0.011 ± 0.020	0.039 ± 0.013	Gy^−2^
	*a* + *c*	0.034 ± 0.062	2.509 ± 1.267	h^−1^
DNA-TEs for uniform field (UF)	*α*_0_	0.388 ± 0.013	0.022 ± 0.007	Gy^−1^
	*β*_0_	0.081 ± 0.036	0.041 ± 0.013	Gy^−2^
	*a* + *c*	1.684 ± 0.911	1.506 ± 1.347	h^−1^
Intercellular communication	*α*_b_	0.388 ± 0.012	0.041 ± 0.027	Gy^−1^
	*β*_b_	0.031 ± 0.032	0.023 ± 0.007	Gy^−2^
	*δ*	0.617 ± 0.081	0.470 ± 0.094	—

**Figure 3:**
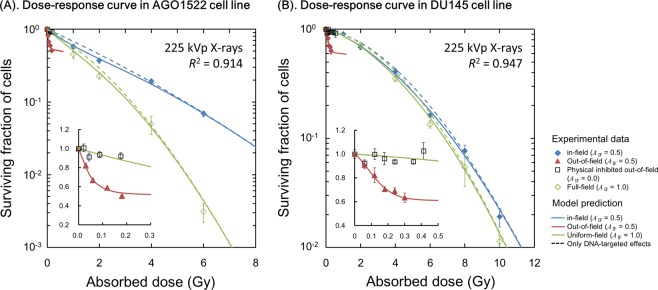
Dose-response curve of cell survival after single-dose exposure. Blue closed diamond, red triangle and green open diamond represent the experimental survival of in-field cells for half-field, that of out-of-field cells for half-field (*A*_IF_ = 0.5) and that of in-field cells for uniform-field (*A*_IF_ = 1.0), respectively. The solid lines are the predicted curve based on DNA-TEs and IC (Eqs (2), (5), (6)), while the dotted lines are the model prediction considering DNA-TEs only (Eq. (2)). The parameters used are listed in Table 1. Experimental data for an exposure with *A*_IF_ = 0 (100% shielded case) is shown as black open squares.

The (*a* + *c*) and *β*_0_ values were deduced from experimental cell recovery curve shown in Fig. 2. The rest of the model parameters were determined by fitting the present model to dose-response curves from single-dose exposures (Fig. 3A,B) via a MCMC simulation. By using the set of model parameters listed in Table 1, cell surviving fractions for the cases of split-dose irradiation, single-dose irradiation and fractionated irradiation were calculated. The (*a* + *c*) = 0.034 ± 0.062 (h^−1^) of AGO1522 cells for modulated field exposure suggests that the SLDR rate is approximately 0, leading to no significant dose-rate dependence.

For AGO1522 cells, the (*a* + *c*) for modulated fields (*A*_IF_ = 0.5) was 0.034 ± 0.062 (h^−1^), whilst that for uniform fields (*A*_IF_ = 1.0) was 1.684 ± 0.911 (h^−1^). The value for uniform fields matches with the reported (*a* + *c*) value^30,35^. However, in AGO1522 cells exposed to a modulated field, the mean value is less than the uncertainty, which suggests no significant dose-rate dependency. The *β*_0_ value is also reduced together with the reduced (*a* + *c*) value, which in the IMK model implies that the probability of two PLLs interacting might decrease or that the number of initial PLL is reduced^35^. In contrast, experimental survival in DU145 cells exhibits the expected SLDR rates^30,35^, i.e., (*a* + *c*) = 2.509 ± 1.267 (h^−1^) for a modulated field (*A*_IF_ = 0.5) and (*a* + *c*) = 1.506 ± 1.347 (h^−1^) for a uniform field (*A*_IF_ = 1.0). Therefore a significant dose-rate dependence was observed for both radiation fields in DU145 cells. The comparison between *in vitro* data and the model suggests that the importance of SLDR in AGO1522 cells exposed to a modulated field is reduced relative to uniform field exposure. Finally, for both cell types there is no significant temporal dependence of out-of-field cell survival, suggesting that there is less impact of SLDR (DNA repair) on out-of-field cell-killing for both cell types (Fig. 2AIII,BIII).

The experimental dose responses for cell surviving fraction are shown in Fig. 3, where Fig. 3A,B are the dose-survival relationship in AGO1522 and DU145, respectively. In Fig. 3, blue closed diamond, red triangle and green open diamond represent the experimental survival for in-field cells under half-field (*A*_IF_ = 0.5), out-of-field cells under half-field (*A*_IF_ = 0.5) and in-field cells under uniform-field exposures (*A*_IF_ = 1.0), respectively. As shown in Fig. 3, the cell viability under half-field exposure (*A*_IF_ = 0.5) is higher than that under uniform-field exposure (*A*_IF_ = 1.0). The smaller colonies measured after 10 Gy uniform-field exposure relative to those measured after half-field exposure are shown in Supplementary Information III (“*Clonogenicity of In-Field Cells Under Modulated-Field Exposure*”).

Based on these trends, we applied the parameter sets for DNA-TEs *θ* = (*α*_0_, *β*_b_, *a* + *c*) for each uniform-field (*A*_IF_ = 1.0) and half-field (*A*_IF_ = 0.5) exposure to fit full model parameter sets *θ* = (*α*_0_, *β*_b_, *a* + *c*, *α*_b_, *β*_b_, *δ*), which are summarized in Table 1. The predicted cell survival curves for single-dose irradiation are plotted as solid lines in Fig. 3, showing that the present model well reproduces the dose response curves for both in-field cells and the higher cell-killing of out-of-field cells at the same absorbed dose.

It should be noted that the low-energy photons scattered from the lead shielding have the potential to enhance the out-of-field cell death in half-field exposures. This impact of the scattered photons on clonogenicity was therefore evaluated before the modelling approach with IC. As shown in Supplementary Information V (“*Estimation of Impact of Low-Energy Photons on Out-of-Field Cell Survival*”), there is no significant impact of scattered photons, with lower energy, on cell survival curve. In contrast, in the Supplementary Information IV (“*Verification of the IMK model for Intercellular Communication*”), previous data which showed that the out-of-field cell survival increased when cells were treated with a nitric oxide (NO) inhibitor^11^ was well reproduced by the model prediction when IC was inhibited (*δ* = 0). From these results, it is clearly suggested that the decrease of out-of-field cell survival is attributed to signalling effects from in-field hit cells to out-of-field non-hits cells (cell killing induced by NO), which correspond to radiation-induced bystander effects^16–20^.

The reduction of radio-sensitivity for irradiated cells under modulated fields has been recently reported for the endpoints of cell survival^45^ and apoptosis^46^; so called rescue^23^ or protective effects^47^. Our *in vitro* survival data and the present model analysis (Fig. 3) are consistent with these recent reports^22,23,45,46^, showing a significant drop in the ${\beta }_{0}$ term and SLDR (Fig. 2 and (*a* + *c*) values in Table 1) when AGO1522 cells are exposed to modulated fields, leading to increased radio-resistance of irradiated in-field cells compared to uniformly exposed cells (Fig. 3).

### Cell recovery during multi-fractionation

**Figure 4:**
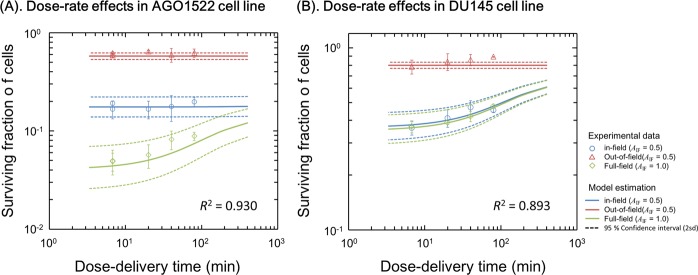
Dose-rate effects on cell survival. The temporal characteristics of the different averaged dose-rates are described in Fig. 1C. Blue, red, green symbols represent the experimental survival for in-field cells, out-of-field cells and uniformly exposed cells. The solid lines represent the mean survival estimated by using model parameters in Table 1, the model formulae of Eqs (1), (5) and (6). The dotted line indicates the 95.4% confidence interval (CI), which is calculated from the MCMC simulation.

To evaluate the reduced importance of SLDR with half-field exposure, we next made a comparison of the model estimation with experimental data for the case of regimens delivered with 4 different dose-rates, i.e., 0.59 Gy/min, 0.20 Gy/min, 0.10 Gy/min and 0.05 Gy/min (Fig. 1C). Figure 4A,B show the comparison between the model estimation and experimental survival data. Blue circle, red triangle and green diamond in Fig. 4A,B represent the experimental data for in-field cells under half-field (*A*_IF_ = 0.5), out-of-field cells under half-field (*A*_IF_ = 0.5) and uniform-field exposed cells (*A*_IF_ = 1.0), respectively. The mean value from the model estimation is described by solid line, and 95.4% confidence interval (CI) is shown by dotted line in Fig. 4. The 95.4% CI was calculated from the uncertainty deduced by the MCMC approach^10^.

From the experiments and modelling, whilst AGO1522 cells after uniform-field (*A*_IF_ = 1.0) exposure exhibit significant cell recovery during dose-delivery, cells exposed to modulated fields see less SLDR and are more radio-resistant (Fig. 4A). In contrast, DU145 cells after uniform-field or half-field exposure exhibit similar recovery kinetics during dose-delivery (Fig. 4B).

**Figure 5:**
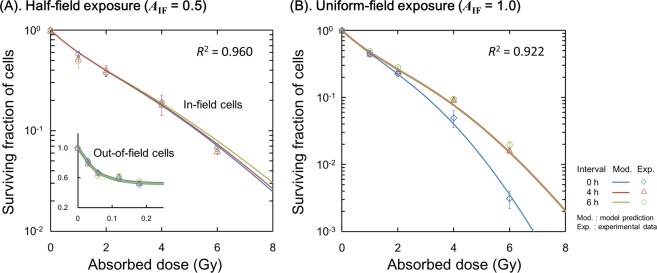
Estimation of cell survival after a split-dose exposure as a function of cumulative absorbed dose. (**A**) is for the case of half-field exposure and (**B**) is for the case of uniform-field, where blue, red, green symbols are the experimental survival for in-field, out-of-field and uniformly exposed cells, respectively. The experimental data were obtained from the previous report by Ghita, *et al*.^13^. The data were modelled using the same procedure used in Fig. 4.

To further validate the different SLDR rate and radio-sensitivity in AGO1522 cells (Figs 2A, 3A and 4A), we added the model predictions for the responses to split-dose irradiation. Figure 5 shows a comparison of AGO1522 cell survival curves estimated by the model (Eqs (1), (5) and (6), Table 1) and experiment data from Ghita *et al*.^13^. As shown in Fig. 5, the different sets of model parameters for half-field and uniform-field are able to reproduce the dose-response curves. This comparison confirms that the different radio-sensitivity created by modulated field results in lower *β*_0_ and (*a* + *c*) values for half-field relative to uniform-field exposures. The implications of this for clinical dose-delivery is summarized in the Supplementary Information VI (*“Application to Clinical Dose-Delivery and Cell Survival Recovery”*), where no statistically significant cell recovery of AGO1522 cells during irradiation using five different treatment modalities is reported.

### Cause of protective effects under half-field exposure

The reason for the difference of in-field cell survival between half-field (*A*_IF_ = 0.5) and uniform-field (*A*_IF_ = 1.0) exposure is still unclear. To better understand the radio-resistance induced by “protective effects”, we attempted to reproduce the survival curve for in-field cells where *A*_IF_ = 0.5 by using the set of model parameters for the uniform field and different response assumptions. Two sets of assumptions were considered: (i) live cells move from the out-of-field region to the in-field region during incubation after irradiation^48^, or (ii) the initial DNA damage yield is reduced under modulated irradiation, which is predicted from the model analysis of *β*_0_ values (Table 1)^42^. From these assumptions, we attempted to determine the processes leading to radio-resistance.

**Figure 6:**
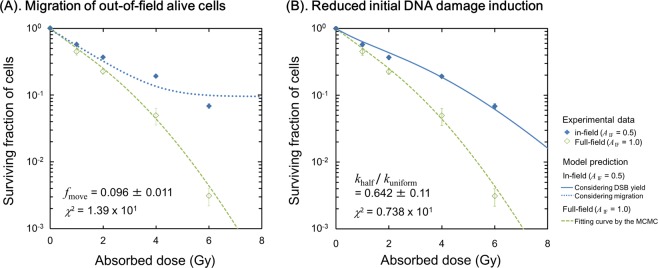
Estimation of the protective effects induced by modulated irradiation. (**A**) Predicted cell survival considering cell migration, in which we assumed that some cells from the shielded population migrate into the in-field area. (**B**) Predicted cell survival considering a reduced the DNA damage yield under half-field exposure. The reduced DNA damage yield is incorporated into the IMK model by using the *k*_half_/*k*_uniform_, where *k*_half_ is the number of PLLs per Gy for half-field and *k*_uniform_ is that for uniform-field.

For cell migration, we assumed a fraction ${f}_{{\rm{move}}}$ of cells move between the in-field and out-of-field regions during incubation after irradiation, and those with no lethal DNA lesions contribute to the surviving fraction. Thus, the measured in-field surviving fraction considering the two cell populations can be given as,

10 $$S=(1-{f}_{{\rm{move}}})\,{S}_{{\rm{IF}}}+{f}_{{\rm{move}}}\,{S}_{{\rm{M}}},$$

where *S*_IF_ is the surviving fraction for the in-field cells which were originally present during irradiation, *S*_M_ is the surviving fraction for the surviving out-of-field cells which have migrated from the out-of-field area (*S*_M_ = 1.0), and *S* is the total surviving fraction of the combined cell population. Applying the model (Eqs (2), (5), (6) and (10)) to the experimental in-field survival data, we obtained *f*_move_ = 0.096 ± 0.011 as shown in Fig. 6A. The model predicts that 9.55% of the surviving out-of-field cells migrate to the in-field area, giving rise to a roughly constant cell survival in high-dose range above about 6 Gy (blue dotted line in Fig. 6A). However, there is no experimental evidence for this, at least *in vitro*. For example, Butterworth, *et al*. performed clonogenic assays quantifying the out-of-field cell survival at high dose (35 Gy in-field dose) and indicated that there were no colonies surviving in the in-field region^11^. Overall, this suggests that migration is not a major factor in the induction of radio-resistance.

Next, we estimated the cell survival curve based on modifying the initial DNA damage yields. Previous DNA damage studies showed a smaller number of DNA lesions (detected by 53BP1 foci) after half-field exposure (*A*_IF_ = 0.5) than the uniform-field exposure (*A*_IF_ = 1.0)^13,42^. We incorporated this reduced DNA damage yield into the dose coefficients (*α*_0_, *β*_0_) in the DNA-TEs model^35^ according to the equation:

11 $${\alpha }_{0}=\frac{ak}{c}\,{\rm{and}}\,{\beta }_{0}=\frac{b{k}^{2}}{2c},$$

where *a* is the rate constant for a PLL to transform into a LL (h^−1^), *b* is the rate constant for two PLLs to interact and transform into a LL and *c* is the rate constant for PLL to be repaired. The experimental DNA damage yield ratio, i.e., 0.642 ± 0.110 (=*k*_half_/*k*_uniform_)^42^ modifies the survival curve of the in-field irradiated cells to agree well with experimental data (blue solid line in Fig. 6B) with a smaller *χ*^2^ value than the model based on cell migration. Overall, as interpreted from the reduced *β*_0_ value (Table 1), we concluded that the reduced initial DNA damage induction due to IC might play a key role of inducing radio-resistance (protective effects) after modulated-field exposure.

### DNA damage mediated cell-cycle effects

**Figure 7:**
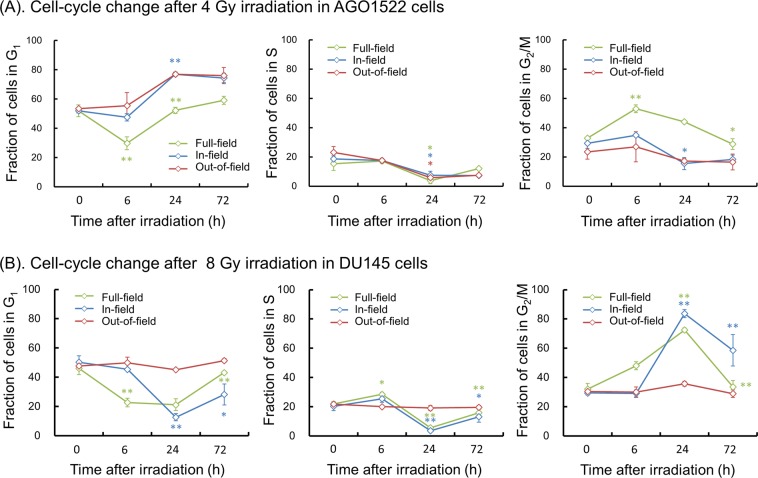
Cell-cycle kinetics after uniform and half field radiation exposure: (**A**) Measured cell-cycle changes after the exposure of AGO1522 cells, (**B**) for DU145 cells. Blue and red symbols represent the fractions of the in-field and out-of-field cells following half-field exposure (*A*_IF_ = 0.5), whilst the green symbol represents uniformly irradiated cells (*A*_IF_ = 1.0). * and ** indicate 5% and 1% significant differences from the previous cell-cycle fraction (i.e., 0 h and 6 h, 6 h and 24 h, 24 h and 72 h).

Finally, to test the impact of modulated fields on DNA damage mediated checkpoint inhibition, cell-cycle studies were performed. Figure 7 shows the dynamics of cell-cycle distribution (fractions of cells in G_1_, S and G_2_/M phases) after irradiation, where (A) is for AGO1522 cells and (B) is for DU145 cells.

In the AGO1522 cells exposed to 4 Gy with uniform-field (*A*_IF_ = 1.0) (green symbol in Fig. 7A), we observed a significant G_2_ block 6 h after irradiation and a cell accumulation at the G_1_ checkpoint 24 h after the irradiation. Under the half-field exposure (*A*_IF_ = 0.5), the population of in-field cells maintains the original cell-cycle distribution until 6 h after irradiation (blue symbol in left panel of Fig. 7A). In contrast, the out-of-field AGO1522 cells exhibit an accumulation in the G_1_ phase, which might be related to the signal-induced cell-killing. DU145 cells exposed uniformly to 8 Gy are blocked in G_2_ 6 h after irradiation with no G_1_ block observed up to 24 h after the irradiation (green symbol in Fig. 7B). This is because DU145 cells are p53 mutant^49^. However, the same (constant) cell-cycle dynamics were observed in the DU145 cells up to 6 h after irradiation in out-of-field populations.

Cell cycle checkpoints are intrinsically related to the degree of DNA damage induction after irradiation^50,51^. Thus, it is reasonable to assume that the stable cell-cycle distribution after half-field exposure (Fig. 7) results from the reduced DNA damage (Fig. 6B). We also suspected the involvement of NO in protective effects because of its involvement in out-of-field cell killing^21^. An additional cell-cycle study with 100 μM aminoguanidine (AG) as an inhibitor of NO was also performed, however AG, under the conditions tested, does not appear alter the cell-cycle distribution, as described in the Supplementary Information VII (*“Cell-Cycle Study with AG Treatment for Modulated Radiation Field”*). The scenario for cell responses in modulated fields is that: (i) there is a reduced initial induction of DNA damage, (ii) cell-cycle checkpoints are not activated due to the reduced DNA damage, and (iii) this reduced DNA damage leads to an increased radio-resistance (and apparent protective effects). However, DNA damage repair within 30 min after irradiation as well as the bystander cross talk have not been examined here, so further investigation of what causes the reduced DNA damage is necessary.

## Conclusion

In this study, we evaluated the impact of modulated radiation fields on the radio-sensitivity and dose-rate effects in human skin fibroblast (AGO1522) and human prostate cancer (DU145) cells. From the *in vitro* experiments and the model analysis, modulated radiation exposures: (i) reduce the yield of PLLs and the importance of cell recovery (SLDR), and (ii) reduce the radio-sensitivity of in-field cells compared to conventional uniform exposures. These effects can be explained by the reduction of the DNA damage in the modulated field, which may also impact on cell-cycle distribution. However, the biological mechanisms governing this change in initial DNA damage remain unclear, so further studies are necessary to identify the underlying mechanisms.

## Supplementary information

Supplementary data

## References

[1] McGarry CK (2011). Temporal characterization and *in vitro* comparison of cell survival following the delivery of 3Dconformal, intensity-modulated radiation therapy (IMRT) and volumetric modulated arc therapy (VMAT). Phys. Med. Biol..

[2] Garibaldi E, Gabriele D, Maggio A, Delmastro E (2016). External beam radiotherapy with dose escalation in 1080 prostate cancer patients: definitive outcome and dose impact. Panminerva Med..

[3] Axelsson J, Davis SC, Gladstone DJ, Pogue BW (2011). Cerenkov emission induced by external beam radiation stimulates molecular fluorescence. Med. Phys..

[4] Kubo HD, Glasgow GP, Pethel TD, Thomadsen BR, Williamson JF (1998). High dose-rate brachytherapy treatment delivery: Report of the AAPM Radiation Therapy Committee Task Group No. 59. Med. Phys..

[5] Kishan AU, Lee P (2016). MRI-guided radiotherapy: Opening our eyes to the future. Integr. Cancer Sci. Therap..

[6] Dasu A, Toma-Dasu I (2015). Will intrafraction repair have negative consequences on extreme hypofractionation in prostate radiation therapy?. Br. J. Radiol..

[7] Kuperman VY, Ventura AM, Sommerfeldt M (2008). Effect of radiation protraction in intensity- modulated radiation therapy with direct aperture optimization: a phantom study. Phys. Med. Biol..

[8] Elkind MM, Sutton H (1960). Radiation response of mammalian cells grown in culture. I. Repair of X-ray damage in surviving Chinese hamster cells. Radiat. Res..

[9] Elkind MM (1984). Repair processes in radiation biology. Radiat. Res..

[10] Matsuya Y, Kimura T, Date H (2017). Markov chain Monte Carlo analysis for the selection of a cell-killing model under high-dose-rate irradiation. Med. Phys..

[11] Butterworth KT (2011). Out-of-field cell survival following exposure to intensity modulated radiation fields. Int. J. Radiat. Oncol. Biol. Phys..

[12] Trainor C (2012). Cell survival responses after exposure to modulated radiation fields. Radiat. Res..

[13] Ghita. M (2015). Impact of fractionation on out-of-field survival and DNA damage responses following exposure to intensity modulated radiation fields. Phys. Med. Biol..

[14] Butterworth KT (2012). Dose, dose-rate and field size effects on cell survival following exposure to non-uniform radiation fields. Phys. Med. Biol..

[15] McMahon SJ (2012). A Kinetic-Based Model of Radiation-Induced Intercellular Signalling. PloS One.

[16] Nagasawa H, Little JB (1992). Induction of sister chromatid exchanges by extremely low doses of α- particles. Can. Res..

[17] Nagasawa H, Little JB (1999). Unexpected sensitivity to the induction of mutations by very low doses of alpha-particle radiation: evidence for a bystander effect. Radiat. Res..

[18] Morgan WF, Sowa MB (2007). Non-targeted bystander effects induced by ionizing radiation. Mutat. Res..

[19] Prise KM, O’Sullivan JM (2009). Radiation-induced bystander signalling in cancer therapy. Nature Reviews Cancer.

[20] Butterworth KT (2015). Time and Cell Type Dependency of Survival Responses in Co-cultured Tumor and Fibroblast Cells after Exposure to Modulated Radiation Fields. Radiat. Res..

[21] Matsumoto H (2011). Induction of Radioresistance by a Nitric Oxide-Mediated Bystander Effect. Radiat. Res..

[22] Lam RKK, Fung YK, Han W, Yu KN (2015). Rescue Effects: Irradiated Cells Helped by Unirradiated Bystander Cells. Int. J. Mol. Sci..

[23] Chen S (2011). Rescue effects in radiobiology: Unirradiated bystander cells assist irradiated cells through intercellular signal feedback. Mutat. Res..

[24] Bentzen, S. M. & Joiner, M. C. The linear-quadratic approach in clinical practice. In: Joiner, M, van der Kogel, A. J., eds. Basic clinical radiobiology. London: Hodder Arnold; 120–134 (2009).

[25] Brenner DJ (2008). The linear-quadratic model is an appropriate methodology for determining isoeffective doses at large doses per fraction. Semin. Radiat. Oncol..

[26] Hall, E. J. & Giaccia, A. J. Time, Dose, and Fractionation in Radiotherapy. In: Hall, E. J., Giaccia, A. J. Radiobiology for the Radiologist 6th ed. Philadelphia: Lippincott Williams & Wilkins: 378–379 (2006).

[27] McMahon SJ (2019). The linear quadratic model: usage, interpretation and challenges. Phys. Med. Biol..

[28] Hawkins RB (1996). A microdosimetric-kinetic model of cell death from exposure to ionizing radiation of any LET, with experimental and clinical applications. Int. J. Radiat. Biol..

[29] ICRU. Microdosimetry. Report 36. International Commission on Radiation Units and Measurements. Bethesda: MD (1983).

[30] Inaniwa T (2013). Effects of dose-delivery time structure on biological effectiveness for therapeutic carbon-ion beams evaluated with microdosimetric kinetic model. Radiat. Res..

[31] Matsuya Y (2017). Modeling cell survival and change in amount of DNA during protracted irradiation. J. Radiat. Res..

[32] Liu ZF (2007). Effective target size for the induction of bystander effects in medium transfer experiments. Radiat. Res..

[33] Friedland W, Kundrát P, Jacob P (2011). Track structure calculation on hypothetical subcellular targets for the release of cell-killing signals in bystander experiments with medium. Radiat. Prot. Dos..

[34] Matsuya Y, Sasaki K, Yoshii Y, Okuyama G, Date H (2018). Integrated Modelling of Cell Responses after irradiation for DNA-Targeted Effects and Non-Targeted Effects. Sci. Rep..

[35] Matsuya Y (2018). Investigation of dose-rate effects and cell-cycle distribution under protracted exposure to ionizing radiation for various dose-rates. Sci. Rep..

[36] Sato, T. *et al*. Features of Particle and Heavy Ion Transport code System (PHITS) version 3.02. *J. Nucl. Sci. Technol*.1881–1248 Online (2018).

[37] Thompson HF (2017). The impact of hypoxia on out-of-field cell survival after exposure to modulated radiation fields. Radiat. Res..

[38] Hawkins RB, Inaniwa T (2013). A microdosimetric-kinetic model for cell killing by protracted continuous irradiation including dependence on LET I: repair in cultured mammalian cells. Radiat. Res..

[39] Okamoto H (2011). Relation between lineal energy distribution and relative biological effectiveness for photon beams according to the microdosimetric kinetic model. J. Radiat. Res..

[40] Hei TK (2008). Mechanism of radiation‐induced bystander effects: a unifying model. J. Pharm. Pharmacol..

[41] Kundrát P, Friedland W, Friedland W (2015). Mechanistic modelling of radiation-induced bystander effects. Radiat. Prot. Dos..

[42] Trainor C (2012). DNA Damage Responses following Exposure to Modulated Radiation Fields. PLoS ONE..

[43] Sato, T. & Hamada, N. Model Assembly for Estimating Cell Surviving Fraction for Both Targeted and Nontargeted Effects Based on Microdosimetric Probability Densities. *PloS ONE*, e114056 (2014). 10.1371/journal.pone.0114056 PMC4245256 25426641

[44] Date H, Sutherland KL, Hasegawa H, Shimozuma M (2007). Ionization and excitation collision processes of electrons in liquid water. Nucl. Instr. Meth. B..

[45] Adrian G, Ceberg C, Carneiro A, Ekblad L (2018). Rescue Effect Inherited in Colony Formation Assays Affects Radiation Response. Radiat. Res..

[46] Widela M, Przybyszewskib WM, Cieslar-Pobudaa A, Saenkoc YV, Rzeszowska-Wolny J (2012). Bystander normal human fibroblasts reduce damage response in radiation targeted cancer cells through intercellular ROS level modulation. Mutat. Res..

[47] Coates PJ, Lorimore SA, Wright EG (2004). Damaging and protective cell signaling in the untargeted effects of ionizing radiation. Mutat. Res..

[48] Wild-Bode C, Weller M, Rimner A, Dichgans J, Wick W (2001). Sublethal Irradiation Promotes Migration and Invasiveness of Glioma Cells: Implications for Radiotherapy of Human Glioblastoma. Canc. Res..

[49] Ambrosini G (2007). Mouse double minute antagonist Nutlin-3a enhances chemotherapy-induced apoptosis in cancer cells with mutant p53 by activating E2F1. Oncogene.

[50] Bedford JS, Mitchell JB (1973). Dose-rate effects in synchronous mammalian cells in culture. Radiat. Res..

[51] Rühm W (2016). Dose-rate effects in radiation biology and radiation protection. Ann ICRP.

