# Supplementary material for: Intensity Modulated Radiation Fields Induce Protective Effects and Reduce Importance of Dose-Rate Effects
Source: Sci Rep. 2019 Jul 1;9:9483. doi: 10.1038/s41598-019-45960-z (PMC6603191; doi:10.1038/s41598-019-45960-z)
Supplement: Supplementary file 1 — Supplementary data [file 41598_2019_45960_MOESM1_ESM.pdf]

# Intensity Modulated Radiation Fields Induce Protective Effects and Reduce Importance of Dose-Rate Effects

Yusuke Matsuya[1,2], Stephen J. McMahon[3], Mihaela Ghita[3], Yuji Yoshii[4], Tatsuhiko Sato[1], Hiroyuki Date[5] and Kevin M. Prise[3]

[1]Japan Atomic Energy Agency (JAEA), Nuclear Science and Engineering Center, Research Group for Radiation Transport Analysis, Tokai, Ibaraki, Japan
[2]Graduate School of Health Sciences, Hokkaido University, Sapporo, Hokkaido Japan
[3]Centre for Cancer Research and Cell Biology, Queen's University Belfast, Belfast, UK
[4]Biological Research, Education and Instrumentation Center, Sapporo Medical University, Sapporo, Japan
[5]Faculty of Health Sciences, Hokkaido University, Sapporo, Hokkaiddo, Japan

## *Supplementary Information*

### I. *Monte Carlo Simulation for $y_D$ Calculation*

The two Monte Carlo codes[1,2] were used for obtaining the microdosimetric quantity of dose-mean lineal energy $y_D$ for 225 kVp X-rays used in the main paper. To verify the sampling algorithm for calculating $y_D$ value, we conducted a benchmark test comparing with the $y_D$ values by Geant4 simulation (version 4.8.2. p01) and those measured by tissue equivalent proportional counter (TEPC)[3].

We set the diameter of the sampling sphere (domain) to be 1.0 μm, and calculated the $y_D$ values of $^{60}$Co γ-rays, 6MV-linac X-rays and 200 kVp X-rays. The geometries for the photon irradiations are illustrated in Fig. S1, which were the same irradiation conditions as reported previously[3]. By using Particle for Heavy Ion Transport Code System (PHITS ver. 3.02)[1], we calculated the energy spectrum of electrons at 6 mm depth from surface for $^{60}$Co γ-rays, at 10 cm depth from surface for 6 MV-linac X-rays and at 1 mm depth from surface for 200 kVp X-rays, respectively. The cut-off energy was set to be 1.0 keV through the PHITS simulation. The example of energy spectrum for 200 kVp X-rays is shown in Fig. S2A. To reduce the calculation time effectively[4], the phase-space file for VARIAN Clinac 600C (10 x 10) was used as a source of 6 MV photons. Inputting the energy spectrum into the in-house code WLTrack for electrons[2], the energy deposition in a domain was sampled uniformly along electron track as shown in Fig. S2B. It is noted that the cut-off energy of electrons was set to be 1.0 eV in the WLTrack calculation.

According to International Commission on Radiation Units (ICRU) Report 36[5], the dose-mean lineal energy represented by $y_D$ is defined as follows

$$y = \frac{\varepsilon}{l} \tag{S1}$$

$$y_D = \int y d(y) \mathrm{d}y = \frac{\int y^2 f(y) \mathrm{d}y}{\int y f(y) \mathrm{d}y}, \tag{S2}$$

where $y$ is lineal energy in keV/μm, $f(y)$ is the probability density of lineal energy and $d(y)$ is dose distribution of lineal energy. After obtaining the $y$-$yd(y)$ distributions shown in Fig. S2C, we calculated the $y_D$ values for $^{60}$Co γ-rays, 6MV-linac X-rays and 200 kVp X-rays.

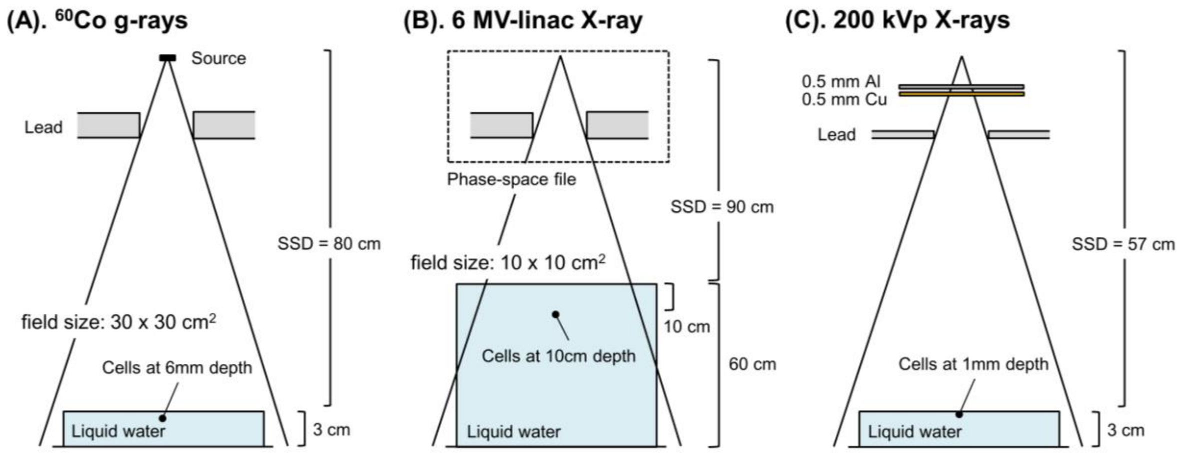


**Figure S1. Illustration of geometries for calculating the $y_D$ values of $^{60}$Co γ-rays, 6 MV-linac X-rays and 200 kVp X-rays.** The geometries were set to be the same as the previous report.[3] Light blue area represents liquid water. The sampling points for $^{60}$Co γ-rays, 6 MV-linac X-rays and 200 kVp X-rays were set at 6 mm depth, at 10 cm depth 1 mm depth from surface, respectively.

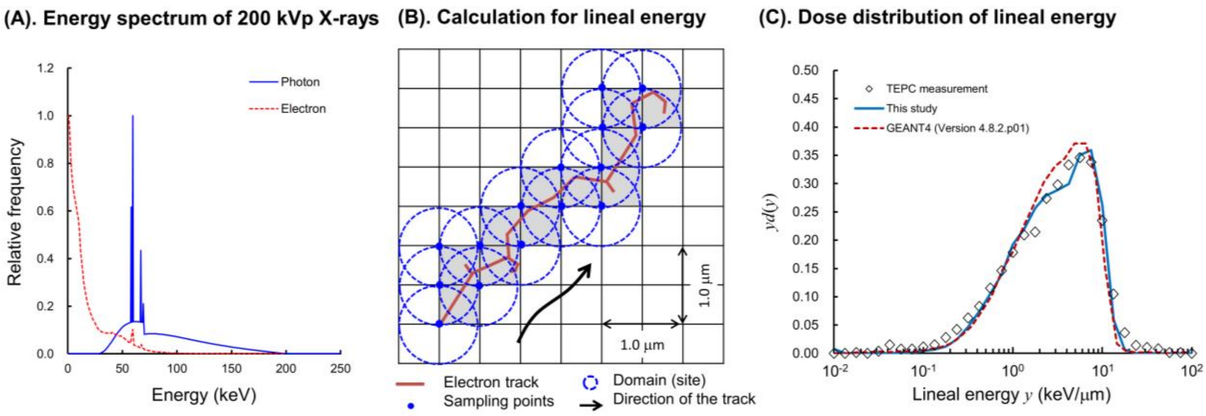


**Figure S2. Procedures for calculating the $y_D$ of photon irradiations.** (A) is energy spectrum of electrons calculated by the PHITS code, (B) is sampling technique of energy deposition in a site, and (C) is the relation between $y$ and $yd(y)$ for 200 kVp X-rays.

| Photon beam type | Dose-mean lineal energy $y_D$ (keV/μm) | | |
|---|---|---|---|
| | This work | Geant4 ver. 4.8.2. p01 | TEPC |
| $^{60}$Co γ-rays | 2.26 ± 0.01 | 2.24 ± 0.01 | 2.34 ± 0.03 |
| 6 MV-linac X-rays | 2.28 ± 0.01 | 2.27 ± 0.08 | 2.36 ± 0.04 |
| 200 kVp X-rays | 4.44 ± 0.04 | 4.41 ± 0.08 | 4.51 ± 0.05 |

**Table S1. Comparison of $y_D$ value among this work, the previous calculation by Geant4 and TEPC measurement.** This work reproduced the photon-energy dependence on $y_D$ value with domain size of 1.0 μm diameter.

As listed in Table S1, the $y_D$ values for three types of photons agreed well with the recommended values by Geant4 simulation and TEPC measurement[3]. As shown in Fig. S2C, the dose distribution of lineal energy ($y$-$d(y)$ relation) calculated in this study coincides with the previous distribution by Gean4 simulation and the TEPC measurement[3]. These results mean that the sampling technique used in this study is precise for calculating the $y_D$ value for photon irradiations.

## II. *Markov chain Monte Carlo for Determining Model Parameters*

The values of $\beta_0$ and $(a+c)$ were determined from the cell survival recovery curve in split-dose experiment (Fig. 2 in the main paper). The rest of cell-specific parameters in the IMK model were determined via a Monte chain Monte Carlo (MCMC) simulation established previously.[6] Here, we summarized the detail of MCMC technique.

The Markov chain Monte Carlo (MCMC) technique provides the probability density function (PDF) of the model parameters.[7,8] This simulation technique is a hierarchical model, and we can obtain the PDF of model parameters following Markov property.[9] In the present model, the prior distribution of $\theta = (\alpha_0, \alpha_b, \beta_b, \delta)$ was set to be normal distribution, with maximum likelihood (ML) value as mean value and reasonable large uncertainty (such as 70% of the ML value) as standard deviation, to obtain the updated posterior distribution with efficient computing performance. Assuming that the uncertainty for –ln $S$ follows the normal distribution, a likelihood function is given by,

$$\begin{aligned} \ell(d\,|\,\theta) &= \Pi_{i=1}^{N_D}\,[\ell(d_i\,|\,\theta)] \\ &= \Pi_{i=1}^{N_D}\left\{\frac{1}{\sqrt{2\pi\sigma^2}}\exp\left[-\frac{(-\ln S_{\mathrm{exp}i}+\ln S_{\mathrm{cal}i})^2}{2\sigma^2}\right]\right\}, \end{aligned} \tag{S3}$$

where $N_D$ is the number of experimental data, $S_{\mathrm{exp}i}$ ($i$ = 1 ~ $N_D$) are the set of experimental survival for the dose $d_i$, $S_{\mathrm{cal}i}$ ($i$ = 1 ~ $N_D$) are those of calculated survival by the present model, and $\sigma$ is the standard deviation of –ln $S$. The ratio of posterior probability for the parameter's candidate (at timing of $t$+1) $\ell(\theta^{\mathrm{candidate}}|d)$ and that for the previous condition (at timing of $t$) $\ell(\theta^{(t)}|d)$ is given by

$$\alpha_{\mathrm{P}} = \frac{\ell(\theta^{candidate}|d)}{\ell(\theta^{(t)}|d)}. \tag{S4}$$

This is the probability ratio to determine the set of model parameters at the next step, so called the transition probability.[9] The algorithm of MCMC used in this study is described in blue flow chart in Fig. S3. By applying the IMK model to experimental cell survival (dose-response) curve after single-dose exposure at 0.59 Gy/min, the set of model parameters $\theta = (\alpha_0, \alpha_b, \beta_b, \delta)$ were determined.

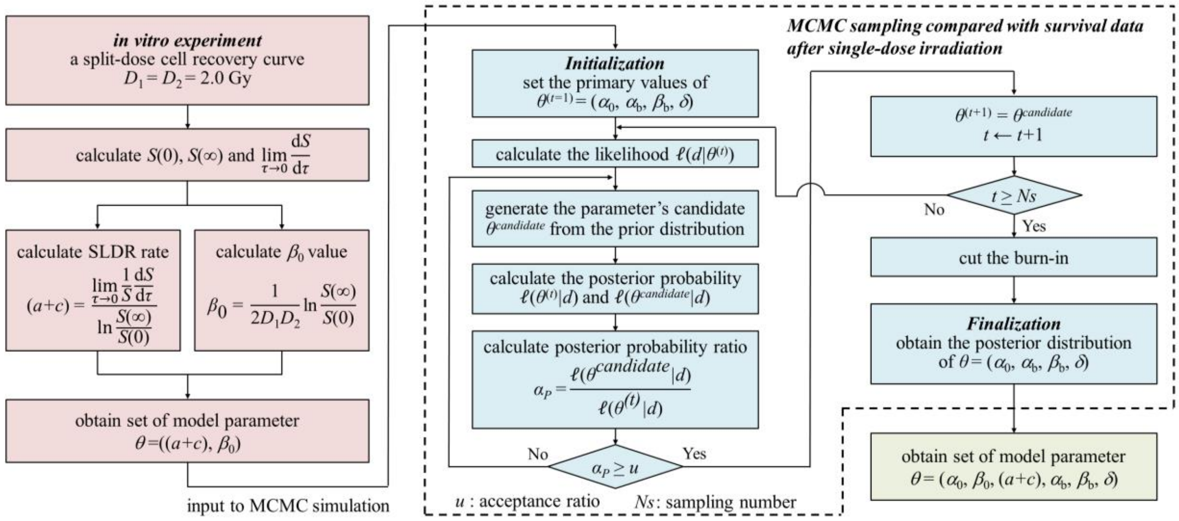


**Figure S3. Procedure to determine model parameters.** Red area of the flow chart represents the deduction of sub-lethal damage repair (SLDR) rate ($a$+$c$) and $\beta_0$ values from experimental split-dose cell recovery curve (see the main paper). Blue area of the chart surrounded by dotted line is algorithm of Markov chain Monte Carlo simulation to determine the rest of model parameters $\theta$ = ($\alpha_0$, $\alpha_b$, $\beta_b$, $\delta$). Following this flow chart, we determined the set of model parameters $\theta$ = ($\alpha_0$, $\beta_0$, ($a$+$c$) $\alpha_b$, $\beta_b$, $\delta$) to demonstrate cell survival after single-dose and fractionated irradiations.

## III. *Clonogenicity of In-Field Cells under Modulated-Field Exposure*

The dose-response curves on cell survival were evaluated by the clonogenic survival assay in the main paper. To be sure that the different radio-sensitivity after half-field exposure, here we show the raw image of the colony formed in this experiment.

Figure S4 shows the raw image of colony formation assay, where A is the colonies of non-irradiated DU145 cells (control group), B is those after 10 Gy exposure under full-field exposure, and C is those after 10 Gy exposure under half-field exposure. Focusing on Fig. S4B and Fig. S4C, the colony size of cells in-field under half field exposure ($A_{\mathrm{IF}}$ = 0.5) is larger than that under uniform-field exposure ($A_{\mathrm{IF}}$ = 1.0).

There are several types of the intercellular signals involved in the cell growth, i.e., transforming growth factor-β1 (TGFβ1).[10] The different size of colony which is linked to the dose-response curve can be explained by the reduced yield of DNA damage induction (Fig. 6B in the main paper). However, the mechanisms to induce the modified DNA damage yield are still unclear. While keeping in mind the increase of cell growth, further investigation for cell responses under modulated fields is necessary in future study.

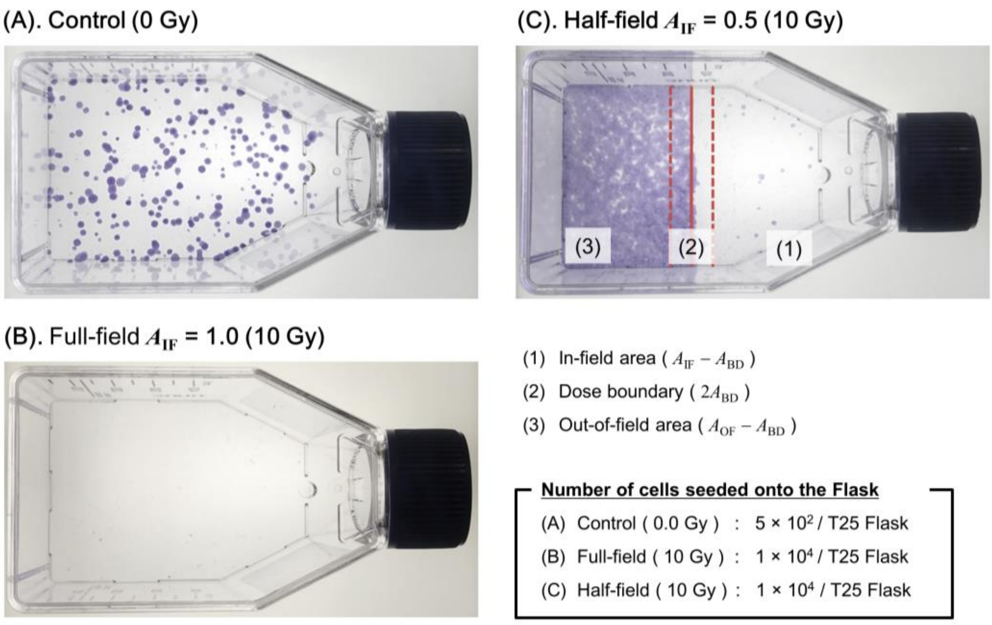


**Figure S4. Image of colonies formed after modulated-field and uniform-field exposure:** A is the colonies of non-irradiated DU145 cells, B is those of the cells exposed to 10 Gy under uniform field, C is those of the cells exposed to 10 Gy under half field. Comparing the colony size formed after uniform-field exposure (Fig S4B) with that of in-field cells after half-field exposure (Fig S4C), it is suggested that the cell viability after half-field exposure is higher than that after the conventional uniform-field exposure.

## IV. *Verification of the IMK model for Intercellular Communication*

In this study, we incorporated the intercellular communication (IC) from in-field hit cells to out-of-field non-hit cells into the cell-killing model so-called "*integrated microdosimetric-kinetic (IMK) model*"[11,12]. In the main paper, we show the formula of cell survival considering DNA-targeted effects (TEs) and IC as the functions of absorbed dose in Gy. Here, we show several additional results for verifying the developed model.

Using model parameters listed in Table 1 in the main paper, we also calculated the dose dependency on out-of-field cell survival based on the model for the cases of constant in-field doses 4.0 Gy or 8.0 Gy (Fig. S5). It is noted that the model formula considering DNA-TEs and IC is given as

$$-\ln S_{\mathrm{T}} = (\alpha_0 + \gamma\beta_0)D_* + \frac{2\beta_0}{(a+c)^2T^2}\left[(a+c)T + e^{-(a+c)T} - 1\right]D_*^2 \tag{S5}$$

$$-\ln S_{\mathrm{NT}} = \delta\left[1 - e^{-(\alpha_{\mathrm{b}}+\gamma_{IF}\beta_{\mathrm{b}})D_{\mathrm{IF}} - \beta_{\mathrm{b}}D_{\mathrm{IF}}^2}\right] e^{-(\alpha_{\mathrm{b}}+\gamma_*\beta_{\mathrm{b}})D_* - \beta_{\mathrm{b}}D_*^2} \tag{S6}$$

$$S_* = S_{\mathrm{T}} \times S_{\mathrm{NT}} \tag{S7}$$

where $S_{\mathrm{T}}$ and $S_{\mathrm{NT}}$ are the surviving fraction of cells for DNA-TEs and IC (non-targeted effects), respectively; ($\alpha_0$, $\beta_0$) for DNA-TEs and ($\alpha_{\mathrm{b}}$, $\beta_{\mathrm{b}}$) for IC are the cell-specific coefficients to dose (Gy) and dose squared ($\mathrm{Gy}^2$); ($a$+$c$) mean the rate of SLDR ($\mathrm{h}^{-1}$); $T$ is dose-delivery time (h); $\gamma$ is microdosimetric quantity given by $\gamma = y_D/(\rho\pi r_{\mathrm{d}}^2)$ in Gy, $\rho$ and $r_{\mathrm{d}}$ are the density of liquid water (1.0

g/cm$^3$) and the radius of a domain considered in the model (set as 0.5 μm); $y_D$ is dose-mean lineal energy in keV/μm; $\delta$ represents the yield of lethal lesions (LLs) in non-hit cells for IC; $D_*$ is absorbed dose in which the symbol * means either in-field denoted as in-field as IF or out-of-field as OF. From the comparison between the model prediction and the experimental data,[13] the present model can flexibly demonstrate the dose-response curve of out-of-field cell survival after the irradiation with constant in-field dose as shown in Fig. S5.

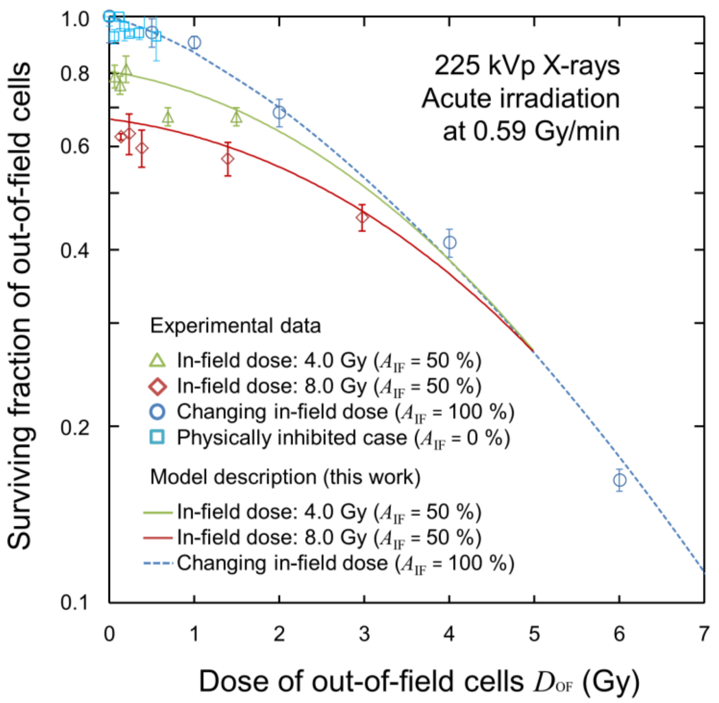


**Figure S5. Model verification for dose-response curve of out-of-field cell survival at constant in-field dose with 4 Gy or 8 Gy in DU145 cell line.** The solid lines were described by using the model proposed in this study (Eqs. (S5)-(S7)), whilst the experimental data (each symbol) is taken from the literature.[13]

In addition, we also compared the model prediction with the experimental data[13] for the case of the treatment with 20 μM aminoguanidine (AG) which is an inhibiter of nitric oxide as one of the intercellular signals. It is noted that $\delta$ value was set to be 0 under the conditions of IC inhibited case (AG+). As shown in Fig. S6, even for the case of AG treatment, the model provides the increment of surviving fraction due to the lack of IC, which suggests that the enhanced cell-killing of out-of-field cells is attributed to IC (defined as radiation-induced bystander effects).

Focusing on the model assumption that the signal effect can cover the entire region of out-of-field under the half-field exposure, the distance for signal delivery is set to be much longer than the previous reports.[14,15] According to the previous report by Koizumi *et al.*, a calcium wave can be detected at distance below 90-100 μm.[14] Meanwhile, apoptosis cells can be observed at distance below ~800 μm.[15] Concerning this discrepancy, the previous studies[14,15] used the three-dimensional tissue model while our study was conducted using cell culture dishes. For this reason, the signals might

move freely in the culture medium after irradiation. Thus, it is necessary in future study to further discuss the signal diffusion distances from the viewpoints of culture dish model and three-dimensional tissue model.

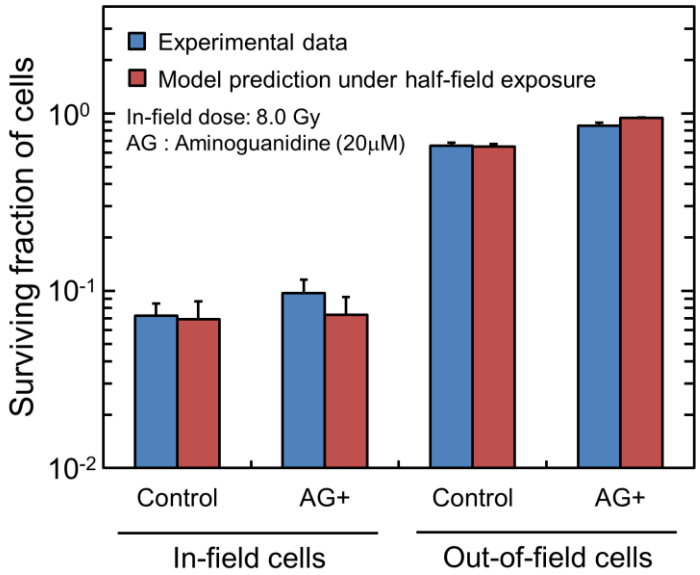


**Figure S6. Model verification for the treatment with 20 μM aminoguanidine (AG) in DU145 cell line.** Red bar represents the model estimation (Eqs. (S5)-(S7)), whilst the blue one is the experimental data taken from the literature.[13]

## V. *Estimation of Impact of Low-Energy Photons on Out-of-Field Cell Survival*

Applying the present model to *in vitro* experimental cell survival in the main paper, we evaluated the impact of modulated radiation field on dose-rate effects and radio-sensitivity (dose-response curve on cell survival). However, the lower energy scattered photons incident to out-of-field cells have a potentiality to affect lager impact on biological effects than the photons incident to in-field cells. So, we also evaluated the impact of the scattered X-rays on cell survival from the standpoint of microdosimery.

As shown in the main paper, the $y_D$ values for in-field cells and for out-of-field cells were 4.393 ± 0.007 keV/μm and 4.769 ± 0.044 keV/μm, respectively. To be sure the radiation quality in out-of-field area, we also calculated the dose-averaged linear energy transfer (LET) by the use of the PHITS code (ver. 3.02)[1], and obtained the values for in-field cells and out-of-field cells were 1.346 ± 0.039 keV/μm and 1.467 ± 0.285 keV/μm, respectively. From the $y_D$ and dose-averaged LET values, it is suggested that the density of energy deposited along the radiation track in lead-shielding area is higher than that in in-field region.

Regarding the calculated $y_D$, we next estimated cell survival curve based on only DNA-targeted effects (TEs) to discuss the impact of scattered photons on out-of-field cells. The formula of cell survival curve for DNA-TEs after irradiation at a constant dose-rate is given by Eq. (S5). According to the previous assumption of the conventional MK model, the parameters of $\alpha_0$ and $\beta_0$ can be fixed

independent of radiation type and the energy[16]. Adopting the assumption, we tried to estimate the dose-response curve by using Eq. (S5) and $y_D$ values for half-field exposure (Fig. 1A in the main paper).

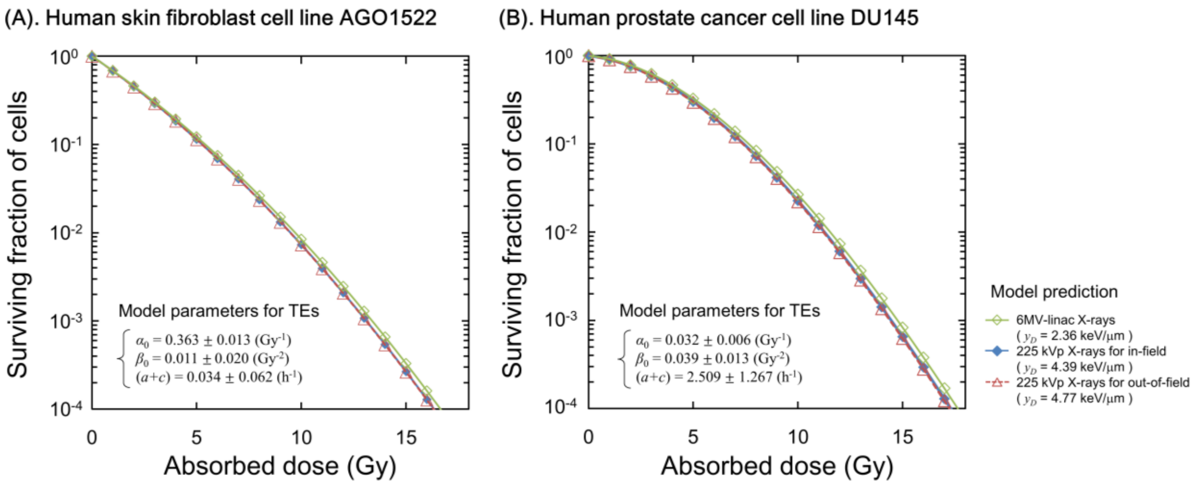


**Figure S7. Estimation of dose-response curve considering only DNA-TEs.** To discuss the impact of scattered X-rays on out-of-field cell survival, we estimated the cell survival using Eq. (S5) and model parameters for modulated exposure listed in Table 1 of the main paper. Figure S7A is the survival curves of AGO1522 cells and Fig. S7B is those of DU145 cells. As shown in these results, there is no difference to enhance cell killing by scattered X-rays for the case of half-field exposure with 225 kVp X-rays.

Figure S7 shows the model estimations of cell survival curve based on DNA-TEs in in-field cells and out-of-field cells. Focusing on the survival curves after irradiation with 225 kVp X-rays in Figs. S7A and S7B, the in-field cell survival curve (blue solid line with closed diamond) approximately coincides with the out-of-field curve (red dotted line with triangle), which means that there is less impact of low-energy photon on the enhancement of cell killing for the case of 225 kVp X-rays irradiation. However, when the $y_D$ value decreases to be 2.36 keV/μm (6MV-linac X-rays), there is subtle difference of cell survival between 6MV-linac X-rays (green solid line with open diamond) and 225 kVp X-rays (the red and blue lines). Thus, the reduction of survival of out-of-field cells is attributed to intercellular signalling from in-field cells to out-of-field cells from this model estimation.

## VI. *Application of Model Prediction to Clinical Dose Delivery*

The reduced importance of sub-lethal damage repair (SLDR) under exposure to modulated fields was suggested in the main paper. To deepen the impact on clinical dose-rate effects, we also tried to estimate cell survival after irradiation with 3 Gy and compared the model estimation with the experimental data reported previously by McGarry *et al*.[21] Upper panels in Fig. S8 shows the temporal characteristics of dose delivery of clinical practices: (A) is accumulated dose delivered to cell population (which corresponds to the in-field dose, $D_{IF}$), (B) is the dose deposited at sub-time interval

of $\Delta T$ (differential dose $D_n$ in the model). Considering the dose delivery in Fig. S8B, we estimated the normalized cell survival after irradiation with 3 Gy following 3D CRT, VMAT (single- and dual-arc) and IMRT (5 and 9-fields) based on the present IMK model. It is noted that the parameters for modulated field (MF) case were used to estimate the cell survival under clinical regimens whilst those for uniform field (UF) case were used for uniform field at constant dose-rate. These parameters are listed in Table 1 in the main paper.

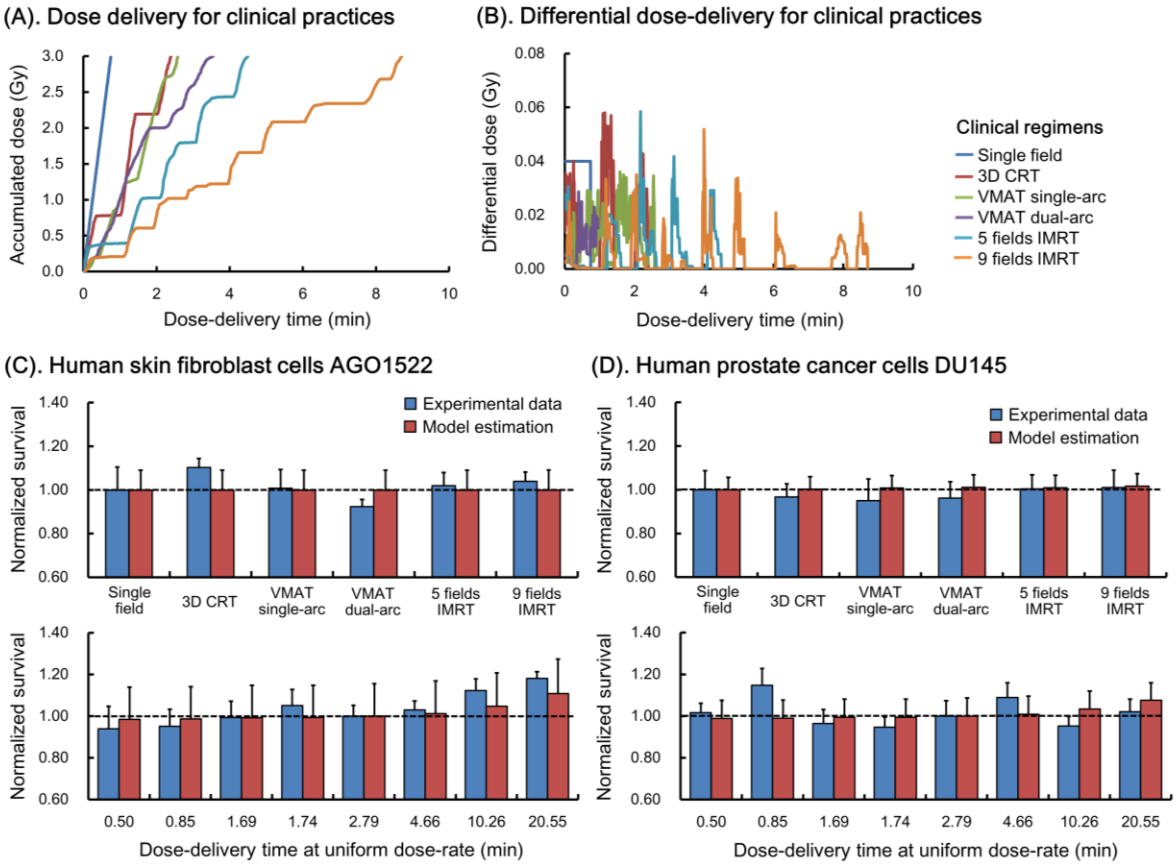


**Figure S8. Comparisons between the model estimation and experimental data for the case of clinical dose delivery.** A illustrates accumulated dose delivered to cell population. B shows the dose deposited at the interval of $\Delta T$ (differential dose $D_n$ in the model). C and D are the comparisons between the model estimation and experimental data of AGO1522 and DU145 cells reported by McGarry *et al*[21]. The error bar represents the 68.2 % confidence interval (CI) of cell survival calculated by MCMC simulation.

Even in case of clinical applications to radiotherapy such as 3DCRT, VMAT and IMRT, the cell survival estimated by the present model considering lower constant rate of SLDR and reduced quadratic-term ($\beta_0$) in AGO1522 exhibits the similar tendency as reported by McGarry *et al.*[20] This comparison means that there is less cell recovery of normal healthy cells (AGO1522) for the cases of clinical dose delivery. In contrast, the model prediction and the experimental data for the case of uniform dose-rate irradiation show a significant recovery of cell survival as dose-delivery time is

protracted. However, the irradiation time to deliver 3 Gy to cells is very short, so further experiments are necessary for check whether or not significance of dose-rate effects is reduced under modulated radiation fields by using high dose.

## VII. *Cell-Cycle Study with AG Treatment for Modulated Radiation Field*

To discuss the reason why the surviving fraction of cells in-field under half-field exposure is higher than that under the uniform-field exposure, we performed flow-cytometric analysis for cell-cycle studies. Here, we show the additional *in vitro* experiments on cell-cycle dynamics in the presence of 100 μM AG for checking whether the NO contributes to protective effects or not[16-19] because the NO-mediated bystander effect can be trigger to cause radio-resistance.[20] As the same manner as the main paper, we focused on the four timing, i.e., 0 h, 6 h, 24 h and 72 h after irradiation.

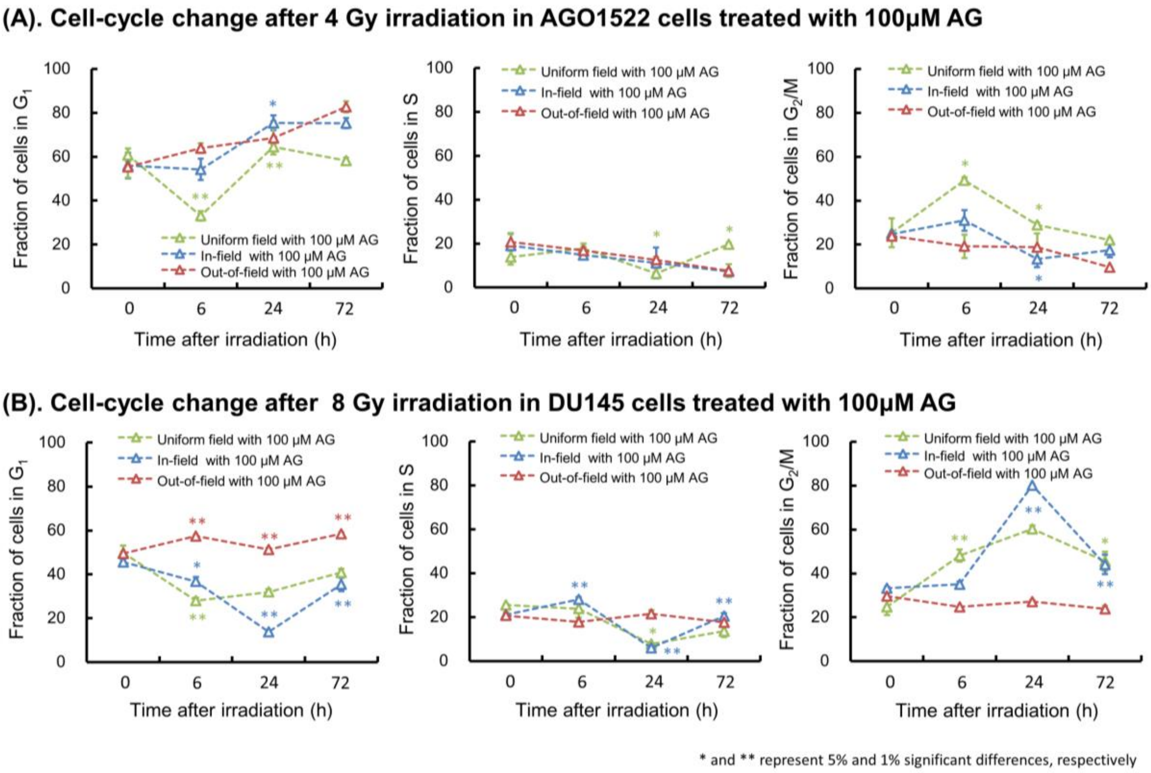


**Figure S9. Cell-cycle kinetics after the exposure in the presence of 100 μM AG:** (A) is the measured cell-cycle dynamics after full- or half-field exposure in AGO1522 cells, (B) is those in DU145 cells. * and ** mean 5% and 1% significant differences from previous cell-cycle fraction (i.e., 0 h and 6 h, 6 h and 24 h, 24 h and 72 h) as the same manner as the main paper. From these results, the inhibitor of NO does not seem to function for deleting the constant $G_1$ fraction until 6h after irradiation.

Figure S9 show the cell-cycle dynamics (change in fractions of cells in $G_1$, S and $G_2$/M phases) after irradiation in the presence of 100μM AG, where (A) and (B) are the AGO1522 cells and the DU145 cells, respectively. As shown in Figs. S9A and S9B, the inhibitor of NO does not seem to function for

deleting the constant $G_1$ fraction until 6h after irradiation. Considering these, the NO might not be the dominant factor to lead to lower radio-sensitivity under half-field exposure in comparison with uniform-field (see Fig. 3 in the main paper). This interpretation is also supported by the results of cell survival shown in Fig. S6, in which the experimental data and the model prediction show the increased cell survival in the presence of the AG. To clarify the underlying mechanisms to lead to radio-resistance (the reduced DNA damage yield under half-field exposure in the main paper), further investigation is necessary.

### Conflict of Interest

The authors declare that they have no conflict of interest.

### Author Contributions

Y. Matsuya, SJ. McMahon and KM. Prise designed the study, Y. Matsuya, M. Ghita performed experiments, Y. Matsuya developed model and calculated cell survival, Y. Matsuya, Y. Yoshii, T. Sato and H. Date calculated dose-mean lineal energy by Monte Carlo codes for transporting the radiation particles, Y. Matsuya and SJ. McMahon wrote the manuscript, KM. Prise supervised the study. All authors reviewed the manuscript.

## V. *References*


1. Sato, T., Iwamoto, Y., Hashimoto, S., Ogawa, T., Furuta, T., Abe, S., Kai, T., Tsai, P.E., Matsuda, N., Iwase, H., Shigyo, N., Sihver, L., Niita, K. Features of Particle and Heavy Ion Transport code System (PHITS) version 3.02. *J. Nucl. Sci. Technol.*1881-1248 Online (2018).
2. Date, H., Sutherland, K.L., Hasegawa, H., Shimozuma, M. Ionization and excitation collision processes of electrons in liquid water. *Nucl. Instr. Meth. B.* 265(2): 515-520 (2007).
3. Okamoto, H., Kanai, T., Kase, Y., Matsumoto, Y., Furusawa, Y. et al. Relation between lineal energy distribution and relative biological effectiveness for photon beams according to the microdosimetric kinetic model. *J. Radiat. Res*., 52(1): 75-81 (2011).
4. Kawrakow, I., Walters, B.R.B. Efficient photon beam dose calculation using DOSXYZnrc with BEAMnrc. *Med. Phys.* 33: 3046–3056 (2006).
5. ICRU. Microdosimetry. Report 36. International Commission on Radiation Units and Measurements. Bethesda: MD, (1983).
6. Matsuya, Y., Kimura, T., Date, H. Markov chain Monte Carlo analysis for the selection of a cell-killing model under high-dose-rate irradiation. *Med. Phys.* 44(10): 5522-5532 (2017).
7. Chib, S. and Greenberg, E. Understanding the Metropolis-Hastings Algorithm. *Amer. Statist.* 49, 327–335 (1995).
8. Gelfand AE and Smith AFM. Sampling-based approaches to calculating marginal density. *J. Amer. Statist. Assoc.* 85, 398–409 (1990).
9. Gelman, A., Carlin, J.B., Stern, H.S. and Rubin, D.B. Model Checking and Improvement. In: Gelman A, Carlin JB, Stern HS and Rubin DB. Bayesian data analysis (vol.2) Boca Raton, FL, USA: Chapman & Hall/CRC; 283–310 (2014).
10. Iyer, R., Lehnert, B.E. Factors underlying the cell growth-related bystander responses to alpha particles. *Cancer Res*. 60: 1290-1298 (2000).
11. Matsuya, Y., Sasaki, K., Yoshii, Y., Okuyama, G., Date, H. Integrated Modelling of Cell Responses after irradiation for DNA-Targeted Effects and Non-Targeted Effects. *Sci. Rep.* 8: 4849 (2018).
12. Matsuya, Y., McMahon, S.J., Tsutsumi, K., Sasaki, K., Okuyama, G., Yoshii, Y., Mori, R., Oikawa, J., Kevin, M.P., Date, H. Investigation of dose-rate effects and cell-cycle distribution under protracted exposure to ionizing radiation for various dose-rates. *Sci. Rep.* 8:8287 (2018).

13. Butterworth, K.T., McGarry, C.K., Trainor, C., McMahon, S.J., O'Sullivan, J.M., Schettino, G., Hounsell, A.R., Prise, K.M. Dose, dose-rate and field size effects on cell survival following exposure to non-uniform radiation fields. *Phys. Med. Biol.* 57: 3197-3206 (2012).
14. Koizumi, S., Fujishita, K., Inoue, K., Shigemoto-Mogami, Y., Tsuda, M., & Inoue, K. $Ca^{2}$ waves in keratinocytes are transmitted to sensory neurons: the involvement of extracellular ATP and P2Y2 receptor activation. *Biochem. J.* 380, 329–338 (2004).
15. Shuryak, I., Sachs, R.K., & Brenner, D.J. Biophysical Models of Radiation Bystander Effects: 1. Spatial Effects in Three-Dimensional Tissues. *Radiat. Res.* 168: 741-749 (2007).
16. Kase, Y., Kanai, T., Matsumoto, Y., Furusawa, Y., Okamoto, H., Asaba, T., Sakama, M., Shinoda, H. Microdosimetric Measurement and Estimation of Human Cell Survival for Heavy-Ion Beams. *Radiat. Res.* 166: 629-638 (2006).
17. Widela, M., Przybyszewskib, W.M., Cieslar-Pobudaa, A., Saenkoc, Y.V., Rzeszowska-Wolny, J. Bystander normal human fibroblasts reduce damage response in radiation targeted cancer cells through intercellular ROS level modulation. *Mutat. Res.* 731: 117–124 (2012).
18. Lam, RKK., Fung, YK., Han, W., Yu, KN. Rescue Effects: Irradiated Cells Helped by Unirradiated Bystander Cells. *Int. J. Mol. Sci.*, 16(2): 2591-2609 (2015).
19. Chen, S., Zhao, Y., Han, W., Chiu, SK., Zhu, L., Wu, L., Yu, KN. Rescue effects in radiobiology: Unirradiated bystander cells assist irradiated cells through intercellular signal feedback. *Mutat. Res.*, 706(1-2): 59-64 (2011).
20. Matsumoto, H., Hayashi, S., Hatashita, M., Ohnishi, K., Shioura, H., Ohtsubo, T., Kitai, R., Ohnishi, T., Kano, E. Induction of Radioresistance by a Nitric Oxide-Mediated Bystander Effect. *Radiat. Res.*, 155: 387-396 (2011).
21. McGarry, C.K., Butterworth, K.T., Trainor, C., O'Sullivan, J.M., Prise, K.M., Hounsell, A.R. Temporal characterization and in vitro comparison of cell survival following the delivery of 3Dconformal, intensity-modulated radiation therapy (IMRT) and volumetric modulated arc therapy (VMAT). *Phys. Med. Biol.* 56: 2445-2457 (2011).
